# The Tomb of the Diver and the frescoed tombs in Paestum (southern Italy): New insights from a comparative archaeometric study

**DOI:** 10.1371/journal.pone.0232375

**Published:** 2020-04-24

**Authors:** Maria Francesca Alberghina, Chiara Germinario, Giovanni Bartolozzi, Susanna Bracci, Celestino Grifa, Francesco Izzo, Mauro Francesco La Russa, Donata Magrini, Emanuela Massa, Mariano Mercurio, Viviana Mollica Nardo, Maria Emanuela Oddo, Stefano Maria Pagnotta, Anna Pelagotti, Rosina Celeste Ponterio, Paola Ricci, Natalia Rovella, Silvestro Antonio Ruffolo, Salvatore Schiavone, Antonio Spagnuolo, Carmela Vetromile, Gabriel Zuchtriegel, Carmine Lubritto

**Affiliations:** 1 S.T.Art-Test di S. Schiavone & C. sas, Niscemi (CL), Italy; 2 Dipartimento di Scienze e Tecnologie, Università degli Studi del Sannio, Benevento, Italy; 3 Istituto di Fisica Applicata “Nello Carrara”, Consiglio Nazionale delle Ricerche (IFAC-CNR), Sesto Fiorentino (FI), Italy; 4 Istituto di Scienze del Patrimonio, Consiglio Nazionale delle Ricerche (ISPC-CNR), Sesto Fiorentino (FI), Italy; 5 Center of Research on Archaeometry and Conservation Science (CRACS), Napoli, Italy; 6 Dipartimento di Scienze della Terra, dell'Ambiente e delle Risorse, Università degli Studi di Napoli Federico II, Napoli, Italy; 7 Dipartimento di Biologia, Ecologia e Scienze della Terra, Università degli Studi della Calabria, Arcavacata di Rende (CS), Italy; 8 Art- Test di E. Massa & C sas, Firenze, Italy; 9 Istituto per i processi Chimico Fisici, Consiglio Nazionale delle Ricerche (IPCF-CNR), Messina, Italy; 10 Department of Analysis and Management of Cultural Heritage, Scuola IMT Alti Studi Lucca, Lucca, Italy; 11 Dipartimento di Scienze e Tecnologie Ambientali, Biologiche e Farmaceutiche, Università degli Studi della Campania “Luigi Vanvitelli”, Caserta, Italy; 12 Energreenup srl, Pietramelara (CE), Italy; 13 Parco Archeologico di Paestum, Paestum (SA), Italy; 14 Istituto nazionale di Fisica Nucleare (INFN) -Sezione di Napoli, Napoli, Italy; Universita degli Studi di Milano, ITALY

## Abstract

The Tomb of the Diver has been subject for many decades of fierce debate among archaeologists and classicists. Since its discovery in 1968, some scholars have considered it a unique example of the lost tradition of Greek painting, others have emphasized Etruscan or Italic parallels. More recently, a possible local production has been suggested. With the aim of trying to solve the archaeological question, an archaeometric comparison among this well-known artwork and several frescoed tombs coming from Hellenistic and Lucan necropolis was carried out. The multi-analytical study was focused on the identification of peculiar features of executive techniques and raw materials since the first period of the archaeological site. The analytical investigation has been preliminary based on a non-destructive approach, performed in-situ by portable equipment including imaging diagnostics and compositional spectroscopic techniques for identifying pigments and the conservation state of original painted surface; subsequently, a further deepening by using destructive techniques was performed in-lab for the mortar-based supports characterization. Archaeometric study suggested that technological choices slightly changed in a time span of about two centuries, highlighting important markers that allow clustering the contemporary artistic productions. Moreover, a comparison with mortars from temples decorations was provided to better understand the whole artistic context. The archaeometric data showed that the Tomb of the Diver could be traced back to a local artisanal tradition and therefore is neither Etruscan nor Greek, but the first and foremost an expression of the local elite culture of Paestum.

## Introduction

**Fig 1:**
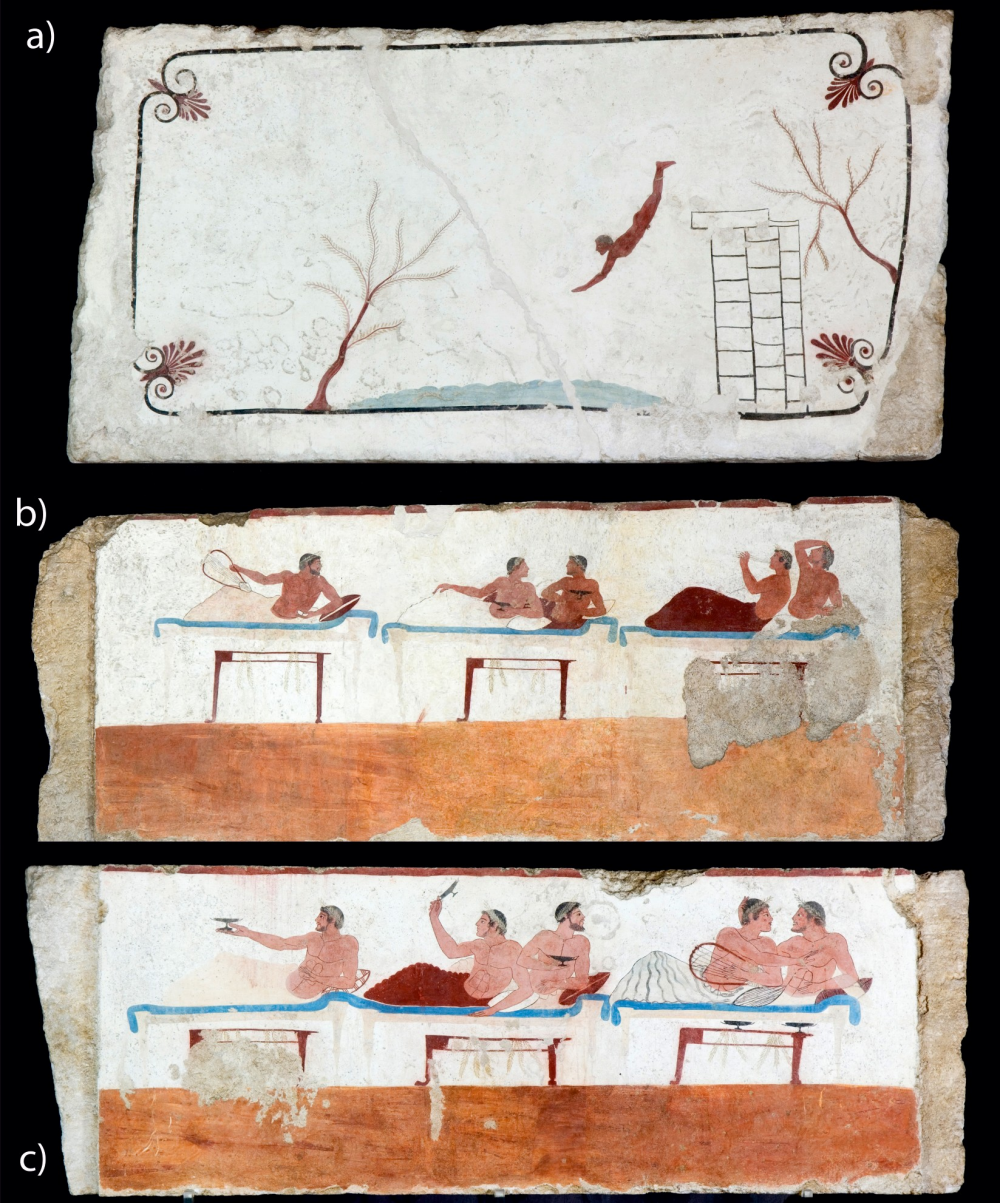
Analysed frescoed slabs of the Tomb of the Diver. (a) Cover slab (215×112×20 cm; TFC). (b) southern slab (225×80×11 cm, TFS). (c) northern slab (244×80×11 cm, TFN).

The Tomb of the Diver is an exceptional painted tomb from the Greek colony of Paestum in southern Italy (Fig 1). It dates to around 500–475 BC and is currently the only tomb with figurative scenes known from a Greek city dating to before the fourth century BC. The paintings are carried out in fresco-technique [1] on a layer of white plaster inside of a stone sarcophagus made of travertine slabs (1.93×0.96×0.79 m) [2]. On the lateral slabs, a Greek banquet (*symposion*) is depicted, while the downside of the lid shows a young man diving into the sea.

**Fig 2:**
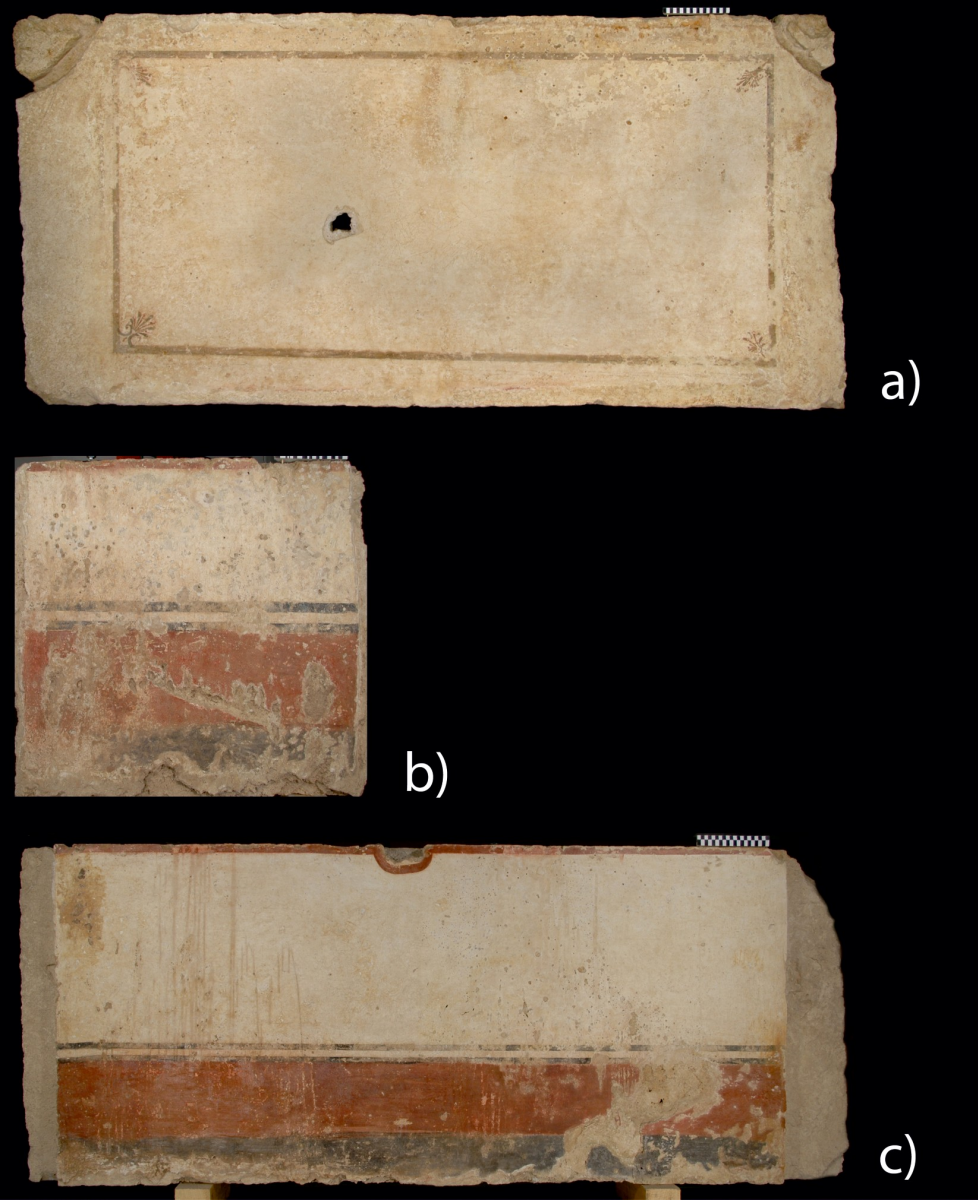
Analysed frescoed slabs of the Tomb of the Palmettes (Tomb Arcioni 781). (a) Cover slab (241×118×20 cm, TPS). (b) Eastern short slab (110×92×10 cm, TPC). (c) Northern slab (240×92×15 cm; TPL).

Since its discovery in 1968, in a small necropolis, 1.5 kilometres south of the ancient city of Paestum, it has been subject of fierce debate among archaeologists and classicists [3]. While some scholars have considered it to be a unique example of the lost tradition of Greek painting, others have emphasized Etruscan or Italic parallels [4]. However, the discovery of other painted tombs around Paestum has raised the question of how the Tomb of the Diver relates to local burial customs and artisanal traditions [5–12]. From the period approximately between 510 and 400 BC, 13 other frescoed tombs are known. Even if there are no figurative scenes, the decoration scheme of most of them recalls that of the Tomb of the Diver, with a red baseboard and a white panel above it. In particular, the lid of the so-called Tomb of the Palmettes (Tomb Arcioni 781) closely recalls the lid of the Tomb of the Diver, as it shows the same frame with four palmettes in the angles (Fig 2). More than a hundred painted tombs are known from the fourth century BC. In this period, figurative paintings become very common.

The present paper presents new archaeometric analysis that shed light on the question of how the Tomb of the Diver relates to other frescoed tombs and great Doric temples from Paestum, starting from the identification of peculiar characteristics of executive techniques and raw materials used in the artworks belonging to the different chronological phases at the Paestum site. In addition to the pigment palettes identification, the present study has examined the whole pictorial stratigraphy and the minero-petrographic features of the mortars from tombs and temples, in order to highlight characteristics of raw materials and painting executive techniques for each archaeological investigated phase.

To date previous scientific studies have been focused on the identification of the used pigments for the pictorial representations on a considerable number of painted tombs. In 1997 H. Brecoulaki analysed different samples of pigments and plasters taken from some tombs, to carry out a scientific study on colours, binders and materials used in Paestum, and in Italy in general, in the pre-roman period [1]. Afterwards, pigments present in pictorial layers of frescoed tombs dated between the end of the 5th century BC and the first quarter of the 3rd century BC (red, yellow, orange, pink, blue, green, black, grey and brown) have been also analysed [13–15]. Ferrari et al. [13] confirm through X-ray fluorescence (XRF) and Raman analyses that the artists' palette is quite limited and coherent with the contemporary funerary art in the Mediterranean basin. Red and yellow ochres prevail on other pigments, whereas the use of Egyptian blue and of a red ochre enriched in manganese is interesting and deeper studies on these materials could be useful to better clarify the commercial background of Paestum during the 4th century BC. Most of the colour hues were obtained by mixing pigments whereas calcite wad added to obtain lighter shades. The authors underline that the choice between one mixture or another could depend both on the availability of raw materials and on stylistic preferences of each workshop [13].

Recently, the archaeometric campaign has been carried out by Italian Association of Archaeometry (AIAr) in collaboration with the Archaeological Park of Paestum. Following an interdisciplinary and multi-analytical approach, the research activity has been focused on: *i)* study of the executive technique; *ii)* characterisation of pigments and organic materials (e.g. pictorial binders and varnishes); *iii)* petrographic study micro-stratigraphic analysis and classification of mortars; *iv)* quali-quantitative characterisation of the mineralogical phases. The scientific investigation has been preliminary based on a non-destructive approach, performed in-situ by portable equipment for characterising the pigments used for decorating the tomb slabs; subsequently, a further deepening on micro fragments of representative samples has been carried out for compositional and stratigraphic analysis of the mortar-based supports for both temples and tombs.

The following scientific methodologies was involved in the present project and carried out by portable equipment: Visible-induced infrared luminescence (VIL); X-Ray Fluorescence analysis (XRF); Fiber Optic Reflectance spectroscopy (FORS); Fourier Transform Infrared Spectroscopy in External Reflectance mode (ER-FTIR); Raman Spectroscopy (RS). Moreover, to guarantee the significance of the results, a preliminary mapping of the surfaces by diagnostics imaging techniques (Infrared reflectography (IRR), UV Fluorescence imaging) was carried out to localise pictorial areas or preparation layers altered by past restorations and to know the conservative interventions that may have changed the original phase of the studied tombs.

In the second phase of the project, in order to acquire the analytical information on the sampled fragments of the mortars the following techniques were employed: Optical Microscopy (OM); Electron Microprobe Analysis coupled with Energy Dispersive Spectroscopy (EMPA-EDS); Attenuated Total Reflection Fourier Transform Infrared Spectroscopy (ATR-FTIR); Simultaneous Thermal Analyses (STA) coupled with FTIR for Evolved Gas Analyses (EGA). Moreover, to guarantee the significance of the results, a preliminary mapping of the surfaces by diagnostics imaging techniques (Infrared reflectography, IRR; UV Fluorescence imaging) was carried out to localise the pictorial areas or preparation layers altered by past restorations and to know the conservative interventions that may have changed the original phase of the studied tombs.

This new integrated analytical approach attempts to provide technical and chemical markers attributable to the artistic production of the first period in order to verify from the archaeometric point of view the belonging of Tomb of the Diver and the coeval Tomb of the Palmettes to a still unexplored local artisanal tradition at the Greek city in 500–475 BC.

### Studied archaeological materials

A multidisciplinary analysis has been carried out on thirteen painted tombs, displayed at museum or stored in warehouses (Figs 1 and 2; S1 Fig), and 2 temples, dated between the end of the 6th century BC and the first half of the 4th century BC, divided in three main chronological phases (i.e. Phase I: ca. 500 BC; Phase II: ca. 400 BC; Phase III: ca. 300 BC) (Table 1). In particular, along with the painted slabs of the Tomb of the Diver, the analyses were carried out on tombs from Arcioni, Gaudo, Andriuolo and Santa Venera necropolis. Moreover, coating mortars of Neptune and Basilica temples were also studied, in order to compare their compositional features with those of coeval tombs.

**Table 1:** List of the tombs and temples studied in the present research, belonged to three different chronological phases.

Tomb	Necropolis	Chronological phase
Tomb of the Diver	Tempa del Prete	Phase I
Tomb of the Palmettes	Arcioni	Phase I
Tomb 210	Gaudo	Phase I
Tomb 314	Gaudo	Phase I
Tomb 76	Andriuolo	Phase II
Tomb 109	Santa Venera	Phase II
Tomb 110	Santa Venera	Phase II
Tomb 6	Andriuolo	Phase III
Tomb 11	Andriuolo	Phase III
Tomb 12	Andriuolo	Phase III
Tomb 20	Andriuolo	Phase III
Tomb 21	Andriuolo	Phase III
Tomb 23	Andriuolo	Phase III
Neptune temple	-	560–520 BC–Phase I
*Basilica*	-	460 BC–Phase I

Phase I (ca. 500 BC); Phase II (ca. 400 B.C); Phase III (ca. 300 BC)

## Methods

The archaeometric study of painted slabs in the first step was based on a non-destructive approach, performed *in-situ* by portable equipment. More than 400 measurements have been performed by using different analytical techniques; measurement areas were selected according to the results of imaging techniques, in an attempt to avoid the restoration areas. To maximize the effectiveness and complementarity of the results, all the analytical techniques used for the project were conducted on the same original pictorial areas previously selected for the identification of organic and inorganic compounds constituting materials.

Moreover, for a deeper characterisation of painted slabs and temples decorations, a second step based on micro-samplings of mortars was conducted, allowing unveiling technological information on the underlying support. An integrated analytical protocol by destructive investigation has been carried out on 30 samples (Table 2). The strategy adopted for analysing samples took into account the amount of available material (it was allowed to collect only a few grams, often not sufficient to perform the entire analytical procedure) and the representativeness of the samples selected for each tomb and temple analysed.

**Table 2:** List of analysed mortar samples collected from tomb slabs stored in the archaeological museum of Paestum and temples preserved in the archaeological site of Paestum, on which destructive analyses have been performed.

ID sample	Tomb/Necropolis	OM	ATR-FTIR	EPMA-EDS	STA
TUF1	Tomb of the Diver/ Tempa del Prete	*		*	
TUF2		*		*	
TUF3		*		*	
TUF4		*		*	
PAL1	Tomb of the Palmettes/ Arcioni 781	*	*	*	*
PAL2		*	*		*
PAL2R		*		*	
PAL2N		*		*	
T11C	Tomb 11/Andriuolo	*	*		*
T11L		*	*		*
T12	Tomb 12/Andriuolo	*	*	*	*
T20	Tomb 20/Andriuolo	*	*		*
T20_1			*		*
T21	Tomb 21/Andriuolo	*	*	*	*
T23	Tomb 23/Andriuolo	*		*	
T76C	Tomb 76/Andriuolo	*	*	*	*
T76L		*	*	*	*
T109C	Tomb 109/Santa Venera	*	*	*	*
T110C	Tomb 110/Santa Venera	*	*		*
T110C_1			*		*
T210	Tomb 210/Gaudo	*	*	*	*
T210L		*	*		*
T314C	Tomb 314/Gaudo	*	*		*
T314L		*	*		*
**ID sample**	**Temple**	**OM**	**ATR-FTIR**	**EPMA-EDS**	**STA**
TN1		*		*	
TN2		*	*	*	*
TN3	Neptune temple	*	*		*
TN4		*	*		*
TN5		*	*	*	*
BS1		*	*		*
BS2	*Basilica*	*	*		*
BS3		*			

### Multispectral imaging analysis

Aimed to obtain a preliminary original and restoration pictorial materials mapping of painted surfaces, multispectral imaging analyses have been carried out by using a high-definition scientific CCD camera characterised by the following technical features: Spectral Sensitivity 0.8–1.1 micron; Quantum Efficiency @ 450, 550, 650, 800, 900, 1000, 1100 nm: 40, 55, 64, 32, 20, 5, 1%; Spatial Resolution 3072×2048 pixel; Reflectogram grey levels: 12 bit/pixel; Large pixel 9x9μm; Full Well Capacity: 100 ke; Dark Current: 0.5 e-/pixel sec; Fill Factor: 100%; Peltier Cooled DT = 40°. The acquisition system makes possible to quantify, store without ageing problems, form data bases and process: calibrate, correct for stray light, form thematic maps, compare, highlight details, etc. In particular, Calibrated UltraViolet fluorescence analysis, with specially filtered UV sources and multispectral acquisition technique, helps to identify the possible existence of inhomogeneity on the surface due to non-original materials, to assess the artwork conservation state and to reveal faded substances or other traces of possible interventions at later stages, guiding us to identify the original materials employed. Moreover, Infrared Reflectography survey allows the study of the deeper layers and in visualising, if existing, the presence or absence of an underdrawing carried out with carbonaceous matter and possibly the technique employed for it, such as tracing of the cardboard, dusting or using a grid.

### Visible-induced Infrared luminescence (VIL)

Two flashes Quantum T5D mounted with B+W 486 UV/IR blocking filter were used for irradiating with visible light the surfaces of painted slabs, in order to explore the peculiar characteristics of some pigments to be luminescent in the infrared region when excited with visible light. The infrared emission was collected with a modified (built–in filter for IR removed) Canon EOS 400D (10.1 Mpixel, CMOS sensor) with Canon lens EFS 28 mm fitted with B+W 093 IR830 infrared filter to cut all stray radiation from visible spectrum and thus collecting only infrared luminescence emission. A white plate Spectralon® (WS-1S-L Labsphere certified standard) and a self-made mock up with Egyptian Blue were used as reference. In this context, such a technique represented a useful tool for the identification and localization of Egyptian Blue pigment [16].

### X-Ray Fluorescence (XRF)

Chemical investigations were performed by using a portable spectrometer consisting of a miniature X-ray tube system, which includes the X-ray tube (max voltage of 40 kV, max current of 0.2 mA, target Rh, collimator 1 or 2 mm), the power supply, the control electronics and the USB communication for remote control; a Silicon Drift Detector (SDD) with a 125 to 140 eV FWHM @ 5.9 keV Mn Kα line Energy Resolution (depends on peaking time and temperature); 1 keV to 40 keV Detection range of energy; max rate of counts to 5.6×105 cps; software for acquiring and processing the XRF spectra. Primary beam and detector axis form an angle of 0 and 40 degrees respectively with the perpendicular to the sample surface.

Tube voltage 35 kV, current 80 μA, acquisition time of 60 s, no filter was applied between the X-Ray tube and the sample, distance between sample and detector around 1 cm are the measurement parameters adopted for this study. The setup parameters were selected to have a good spectral signal and to optimize the signal to noise ratio (SNR).

### Statistical treatment of XRF spectra

Statistical treatment of XRF spectra has been carried out for identifying the main compositional clusters and evaluating eventual differences within the three chronological phases.

Raw data (namely the acquired XRF spectra) were calibrated in order to precisely assign the characteristic 956 emission lines (in KeV) of each chemical element. After the calibration, the data were treated for enhancing the most relevant peaks and removing the noise. We log-transformed each sample x_j_ with j = 1,2,3…956 and got y_j_ = logx_j_. The y_j_ were smoothed with a moving average based on 10-konts with Gaussian weighting system. Then, we considered the residuals z_j_ = x_j_-y_j_ that are clean from the background noise.

To enhance the peaks, we considered the $z_{j}^{*}=z_{j}*I\left(\frac{z_{j}}{s}>\delta \right)$, where *I*(.) is the indicator function, *s* is the standard deviation of the residues, and *δ*>0 adjust the distance with *s*. The removal of background noise forms some islands *I_k_* with $z_{j}^{*}>0$ for every *j∈I_k_*, $z_{j}^{*}$ is equal to zero outside of the islands. The signal peaks *p_k_* are the maximum of the islands.

Such transformation from original counts to maximum of the function was performed for each sample. The peaks in each sample where used to recognize the chemical elements. We restricted the analysis to those elements appearing in at least the 5% of the samples, and no more than 95%.

The final data-matrix (105 samples and 34 chemical elements) contain 0s and 1s, where the 1s means that the element is observed in the samples, while the 0 is the absence of the substance. From the data-matrix we compute the distance matrix with the simple-match distance, and then we performed a hierarchical clustering with the ward’s linkage.

### Fiber Optics Reflectance Spectroscopy (FORS)

Single spot analyses for each pigment were performed using Fibre Optics Reflectance Spectroscopy (FORS), a spectroscopic technique which analyses the light reflected from a surface illuminated with visible light.

FORS measurements were carried out in the spectral range 400–800 nm by using a tungsten lamp (20W) as source and the grating Ocean Optics (model HR2000) as detector. Optical fibre bundles were used both to drive the light on the surface under analysis and to collect the reflected radiation. The measuring head geometry was 45°/0°/45°. The probe, in contact with the surface, was a homemade fibre holder, which, at the same time, guarantees a soft contact and permits to fix the best distance from the surface. This allow to maximize the signal and to maintain the measuring area shielded from undesired external light. The analysed area was 2 mm^**2**^ and each acquired spectrum was the average of 30 scans. As a reference, a Spectralon® plate (WS-1S-L Labsphere certified standard) was used. Spectra were compared with reference ones available in ICVBC-CNR reference database to identify pure pigments or mixture.

### Raman Spectroscopy (RS) and Surface Enhanced Raman Spectroscopy (SERS)

BRAVO Handheld Raman Spectrometer by Duo LASER was used to collect Raman spectra of the tombs in situ. BRAVO uses a patented technology (SSE^™^, Sequentially Shifted Excitation, patent number US8570507B1) [17] to mitigate fluorescence phenomena and it is equipped with two excitation lasers with wavelengths (DuoLaser^™^) centered at 785 and 853 nm [18]. The spectra have been collected in the 300–3200 cm^**−1**^ range with integration times no longer than 60 s. All spectra were collected with OPUS software.

In combination with Raman analysis, SERS spectroscopy has been used for better investigating the possible presence of protective films. This technique exploits the amplification of Raman scattering by molecules adsorbed on a surface of a noble metal. *Ad-hoc* substrates constituted by polishing films in which silver nanoparticles were used for collecting micro-samples. Thin film of AgNps was deposited on flexible polishing sheets coated with an aluminium oxide powder with a rms roughness of 0.3 μm. Spectra have been acquired with Raman Jobin Yvon HR 800 spectrometer using a He–Ne excitation laser radiation of 632.8 nm.

### Fourier Transform Infrared Spectroscopy (FTIR)

Surfaces of painted slabs have been analysed at room temperature by means of a Bruker Optics Alpha-R portable spectrometer with an External Reflectance (ER) module for contactless and non-destructive analyses, covering a circular area of about 3 mm of diameter [19].

The instrument is equipped with a ROCKSOLID^**TM**^ interferometer and a ZnSe/KBr beam splitter with a DTGS detector. The spectra were collected in a spectral range between 4000 and 400 cm^**-1**^, with a resolution of 4 cm^**-1**^ and 128 scans for each run (ca. 2 minutes). Bruker Opus 7.2 software was used for data acquisition and processing. The spectra were treated by baseline correction, smoothing and log-transformation (A’ = log (1/R)).

Mortars were, instead, analysed by means of Attenuated Total Reflectance module (ATR), equipped with a diamond crystal, at same resolutions and spectral range, using 64 scans for each run.

### Optical Microscopy (OM)

The transmitted light optical microscopy investigations (OM), performed by using a Zeiss Axiolab associated with AxioCam MR for digital image acquisition, were aimed to identify the mineral-petrographic characteristics of the mortars, in terms of both binder and aggregate typology; to highlight the stratigraphic relationships between the layers; determine the conservation state of original materials and any contribute due to previous restoration.

### Electron Microprobe Analysis and Energy Dispersive Spectrometry (EMPA-EDS)

Electron microprobe analysis (EMPA) coupled with energy-dispersive spectroscopy (EDS) were performed on 14 micro samples (Table 2) in order to obtain quantitative data and to characterise the binder chemical composition in the mortars, verifying any executive difference along the three chronological phases. The technical characteristics of the used JEOL JXA 8230 instrument are: Source: W/LaB6; 5 Spectrometers WDS with LDE, TAP, PETJ, LiF, crystals; Proportional Counter Detector, Xe-Filled Detector; 5th spectrometers with large crystals: PETL and LIFL for trace element analysis; 1 Spectrometer EDS–JEOL EX-94310FaL1Q - Silicon drift type (Resolution: 129 eV; Window: Ultra-Thin Window (UTW); Detection area: 10mm^2^).

The EMPA images were acquired according to the following instrumental parameters: HV: 15 KeV; probe current: 10 nA; working distance: 11mm; Image: BSE–SE signal; detector image: Solid State detector (SSD), Everhart Thornley detector (SE); Image size: 2560 x 1920 pixel. Moreover, the EDS analyses were carried out according to the following measurement parameters: 15 keV HV; 10 nA probe current; 11mm working distance; 40° take off; and 30 seconds live time.

The measurements were performed after coating the sample with a thin and highly conductive film ultra-pure graphite (± 5 nm thickness, applied by Sputter—Carbon Coater QUORUM Q150T-ES, 70 A pulse current and 2.5 sec pulse time).

### Simultaneous Thermal Analyses (STA) coupled with Evolved Gas Analysis (EGA)

Thermogravimetric (TG) and Differential Scanning Calorimetric (DSC) analyses were simultaneously performed using a Netzsch STA 449 F3 Jupiter thermal analyser coupled with a Bruker Tensor 27 for Evolved Gas Analysis (EGA) in FTIR. Powdered samples (20–30 mg) were placed in alumina crucibles and heated from room temperature up to 1050°C (10°C/min heating rate) in ultra-pure air atmosphere. The FTIR spectra were acquired using 8 cm^*-1*^ resolution, 32 spectra scans per minute, 100 spectra scans for background, in the spectral range 4000–600 cm^*-1*^. Netzsch Proteus 6.1.0 and Opus 7.0 software were used for data analysis.

## Results

### Pictorial layer

#### Surface treatments and alteration products

In order to guarantee a correct identification of original pictorial materials, vibrational spectroscopic analyses (ER-FTIR, SERS) highlighted the spectral responses of the surface treatments due to previous restoration works and weathering products affecting the painted slabs.

**Fig 3:**
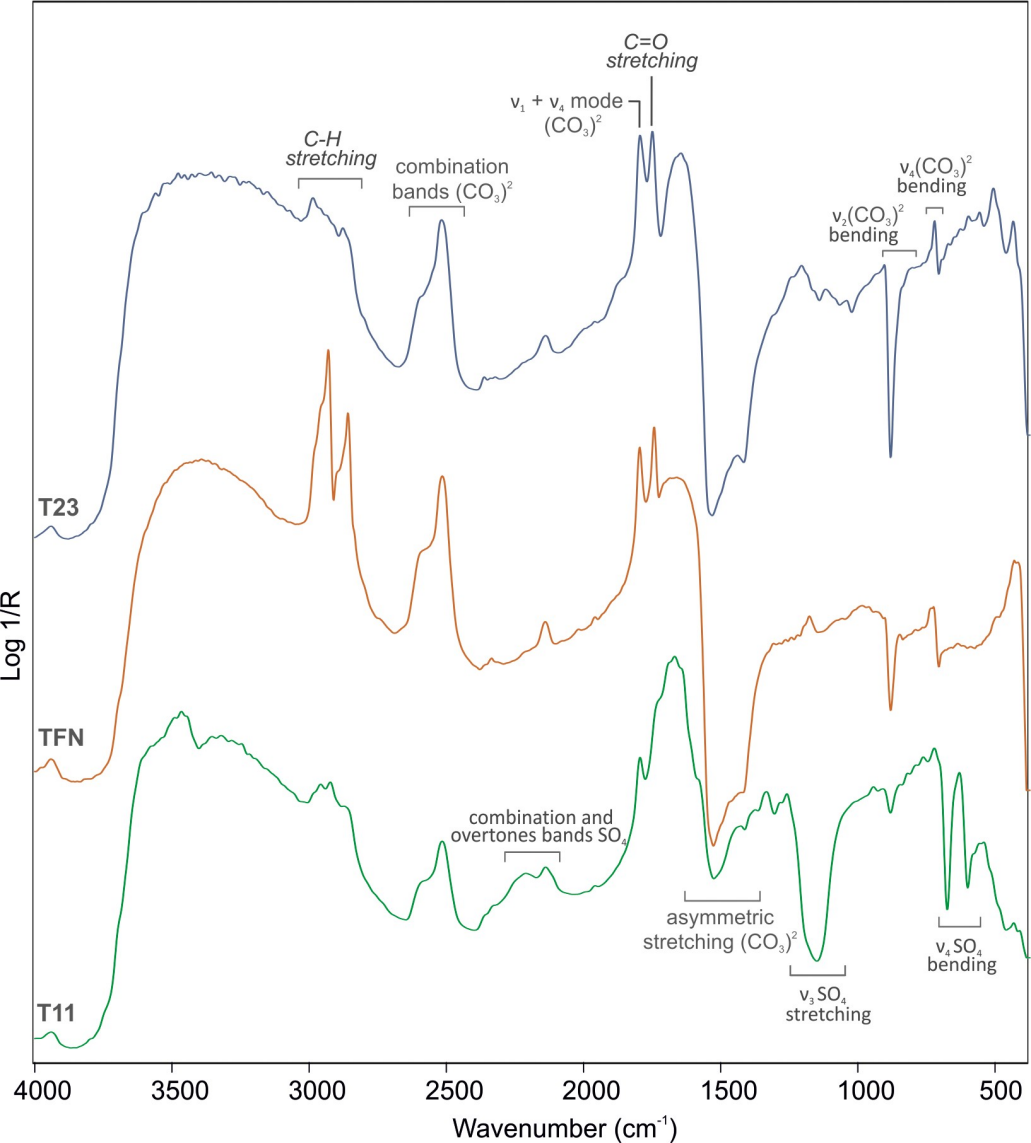
Representative ER-FTIR spectra. T11: Tomb 11, TFN: Tomb of the Diver, northern slab, T23: Tomb 23. Infrared bands of organic compounds are reported in italics.

**Fig 4:**
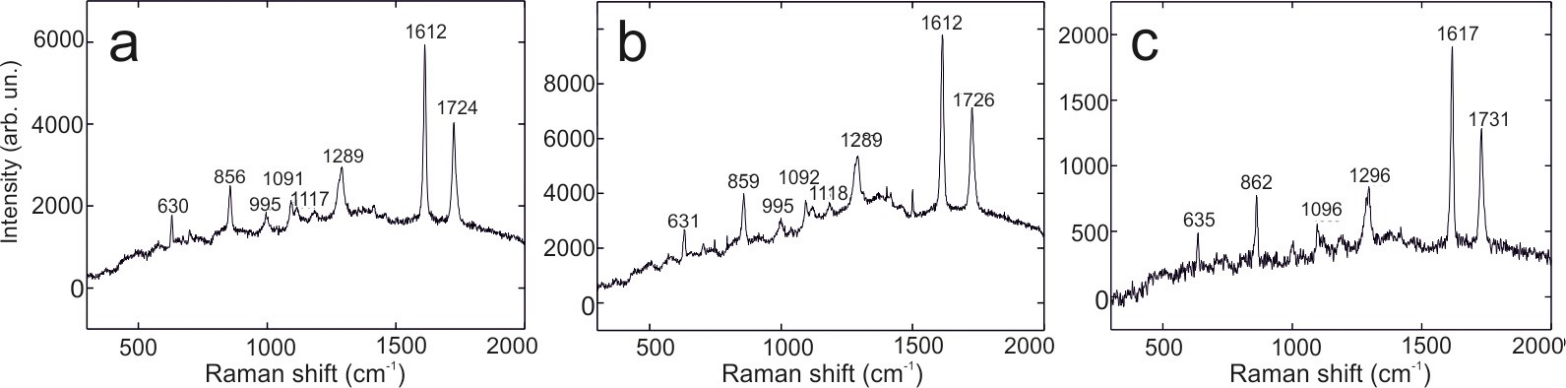
SERS spectra of nano-samples. (a, c) Tomb of the Palmettes. (b) Tomb of the Diver.

ER-FTIR spectra, in fact, revealed the typical signatures of organic materials at ca. 2982, 2953, 2926, 2870 (C-H stretching vibration) and 1740 cm^*-1*^ (C = O stretching vibration) (Fig 3). Although weak and not well-defined bands were observed, it was possible to identify the use of an acrylic resin [20–24]. The occurrence of such product was further confirmed by SERS analyses on polishing film substrates taken on the Diver and Palmettes tombs (Fig 4), in which Raman signatures at ca. 1730, 1615, 1296, 1117, 1091, 995, 860 and 630 cm^*-1*^ were observed [17].

It should be noted that on the Tomb T11 and on the Tomb of the Diver sharper peaks at ca. 2960, 2930 and 2850 cm^*-1*^ (C-H stretching vibrations) were observed (Fig 3), suggesting the presence, along with the acrylic resin, of another organic compound, whose C-H bands were affected by derivative-like features attributable to its strong absorption coefficient. Comparisons with available literature [25,26] suggested that such features are consistent with the presence of wax.

**Fig 5:**
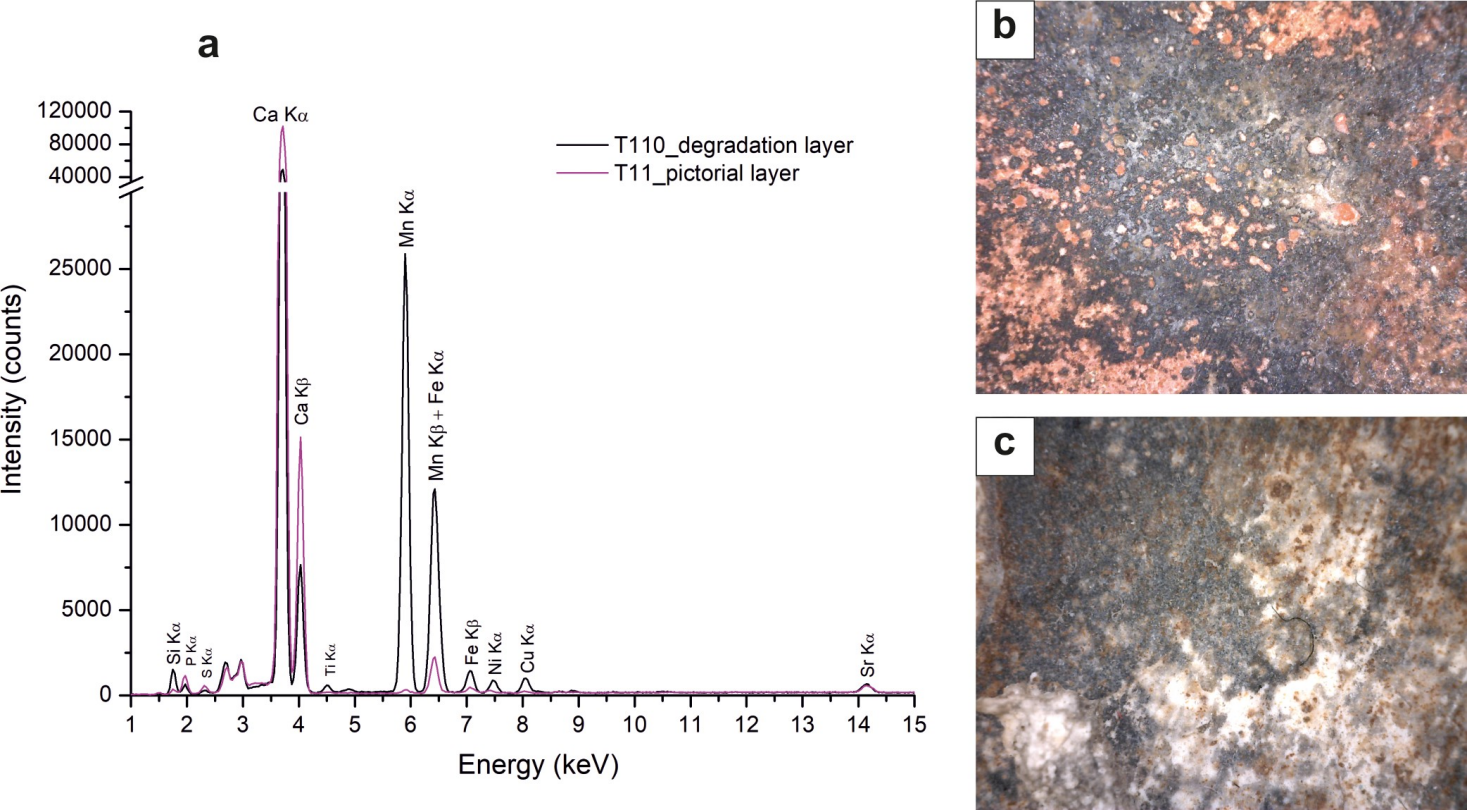
Black patinas containing manganese and black pictorial layers. a) XRF spectra of black degradation layers acquired on the pictorial surface of the painted tomb T110 enriched in manganese, compared with a representative spectrum of black pictorial layers (tomb T11); digital microscope images acquired on black layer of T110 (b) and T11 (c) respectively.

As alteration products, ER-FTIR and Raman spectroscopies detected the typical features of sulphates. Actually, on the tomb T11 infrared broad bands at ca. 2230 (2ν_*3*_ SO_*4*_; ν_*2*_ +ν_*L*_ H_*2*_O) and 2140 cm^*-1*^ (ν_*1*_ + ν^*3*^ SO_*4*_) along with sharp peaks at ca. 1150 (ν_*3*_ antisymmetric SO_*4*_ stretching vibration modes), 670 and 600 cm^*-1*^ (ν_*4*_ antisymmetric SO_*4*_ bending vibration modes) are indicative of the presence of gypsum (Fig 3), as confirmed by the peak at ca. 1008 cm^*-1*^ in Raman spectra [27]. In some cases, ER-FTIR spectra also show a weak band at ca. 1320 cm^*-1*^, likely due to the presence of oxalate, a weathering product whose crystallisation is attributed either to biological attacks of the surfaces (e.g. from lichen, fungal, algal) or to the degradation of organic materials (e.g. wax, animal lipids, eggs) added as surface treatments [28]. Moreover, on the tombs 109, 110, 20, 23 and 76 (S1 Fig) superficial black patinas were observed. XRF spectra revealed the presence of manganese (Fig 5A), likely due by the activity of manganese oxidizing microbes during the burial period [29]. This deterioration patinas (Fig 5B), observed on tomb slabs preserved in the deposits and not yet subjected to a complete cleaning intervention, show evident differences in chemical composition with the unaltered black pictorial layers, displaying an enrichment in manganese, silicon, and iron content. With respect to the black pigments (Fig 5C), they cover the painted slabs, hiding the underlying pictorial layers.

#### Pigments identification

For each pigment hue, XRF, Raman, FORS and ER-FTIR analyses was carried out on original pictorial layers. Below the results obtained from the whole set of analytical techniques used for *in situ* measurements are reported.

*White pigments*. The calcite is the main mineral constituting the white layers of the whole set of analysed tombstones, as demonstrated by the detection of Ca by XRF, the typical Raman peaks at 712 and 1086 cm^-1^ and infrared bands at ca. 2510 (with a shoulder at ca. 2590 cm^−1^), 1794 cm^-1^, *Reststrahlen* and derivative effects at ca. 1410, 873 and 713 cm^−1^. Only the Raman spectrum acquired on T11 slab also shows an additional band at 1008 cm^-1^ attributable to the gypsum, likely derived from sulphation phenomena affecting the slab [30,31].

The obtained results are supported by several studies carried out on the frescoes of other Campania archaeological sites, which confirm the use of calcite as the most widespread white pigment [32] along with other carbonates [33,34]. Carbonates are widely available in Campania territory; they have always represented an important georesource not only as white pigment but also for the production of lime-based mortars [35]. Moreover, micro-stratigraphic evidences showing the absorption of pigment in the pictorial layer (see the paragraph *Textural*, *mineralogical and compositional features of mortars*) and the presence of calcite as the sole binder detected in the paint layers of slabs suggest the execution of decoration by using the fresco technique executed by mixing pigments with slaked lime and water and applying on a thin layer of lime-based plaster while this was still moist. [26,36].

**Fig 6:**
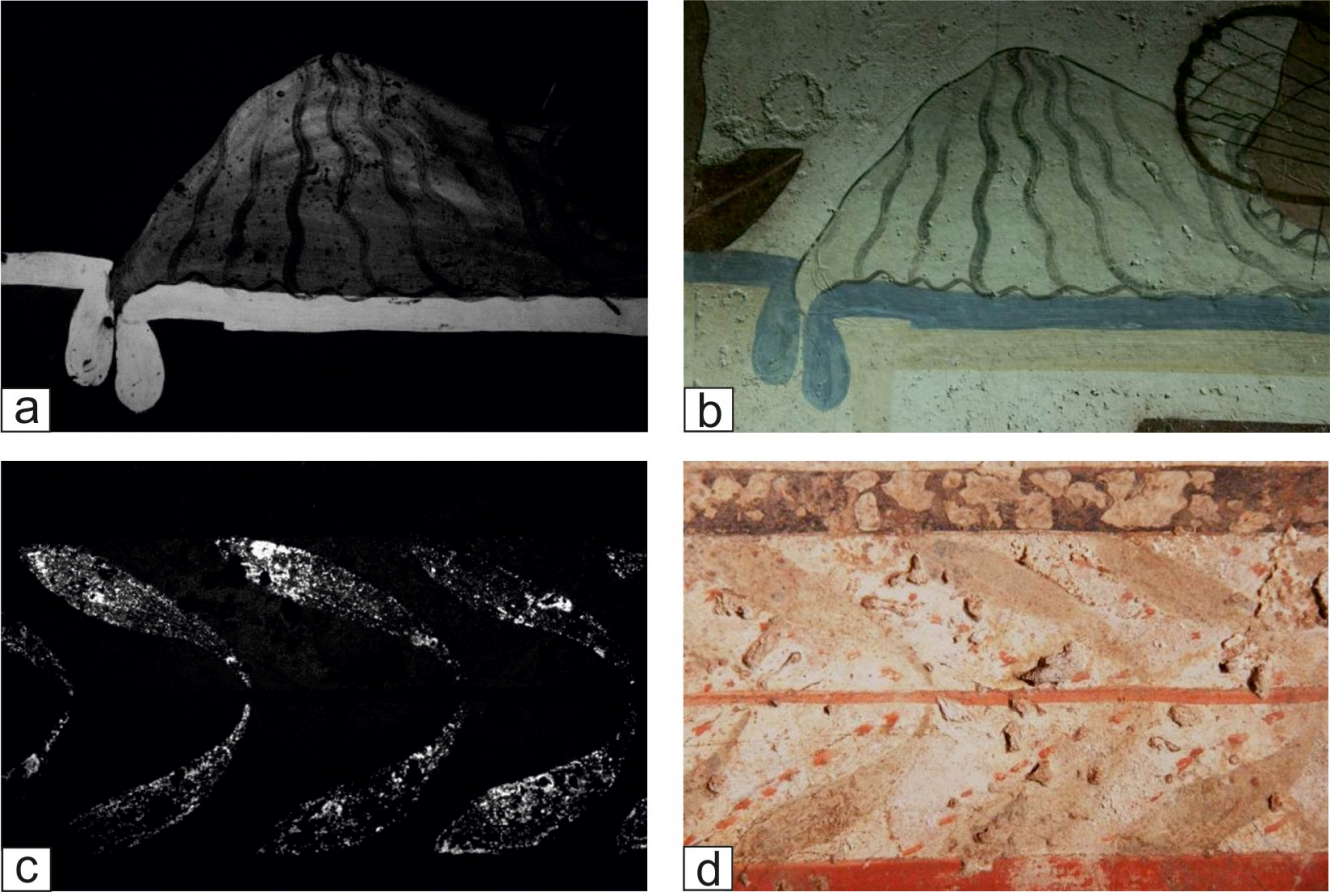
VIL and visible images of blue and green decorations. (a) VIL image of a northern slab detail of the Tomb of the Diver. (b) Corresponding visible image of the northern slab detail of the Tomb of the Diver. (c) VIL image of the green decoration of the Tomb 21. (d) Corresponding visible image of the green decoration of the Tomb 21.

*Blue pigments*. The light blue layers were investigated in the painted slabs belonging to the Tomb of the Diver and tomb T6. VIL images allowed to identify immediately the presence of Egyptian Blue as constituent of the light blue pictorial layers localised on the two tombs belonging to different periods, the Tomb of the Diver (Fig 6A and 6B), and tomb T6. All the VIL positive blue layers have been analysed by spectroscopic techniques in order to carry out the univocal identification of this blue pigment. XRF generally detected the presence of copper, along with calcium and lower amounts of aluminium, silicon, iron and strontium. The presence of copper confirms the use of Egyptian blue, a synthetic pigment constituted of cuprorivaite blue crystals (CaCuSi_4_O_10_ or CaOCuO·(SiO_2_)_4_—calcium copper tetrasilicate) and obtained by the mixture of silica sand, lime, compounds of copper (or copper mineral fragments) and a fluxing (soda or plant ash) [37–39]. Finally, the presence of Egyptian blue has been systematically and definitely confirmed by FORS analyses. The detected absorptions in all FORS spectra are related to electronic transitions attributable to Cu^2+^ ions, responsible of the photoluminescence of the cuprorivaite. On the Tomb of the Diver, Egyptian Blue was not only used for painting blue areas but also to create different shades. In particular, on the north slab the vest of the second person from right, presents some Egyptian blue even if in visible light the vest appears almost white with some black lines (Fig 6A and 6B). In this case, the little amount of the blue pigment was intentionally used to impart a colder hue to the vest.

In Campania region, attestations of Egyptian blue production have been found in Cuma and Pozzuoli [32,37,38]. Differently from the pigment palette of the Tomb of the Diver where the presence of Egyptian blue was clearly noticed, for most of the Paestum tombs dated between the end of the 5th century BC and the first quarter of the 3rd century BC the utilization of this pigment appeared unusual, as also discussed in previous literature [13]. This evidence could depend on the not so easy production or commercial availability of Egyptian blue in this period, which limit its use just for the tombs of the most important or wealthier families [13].

**Fig 7:**
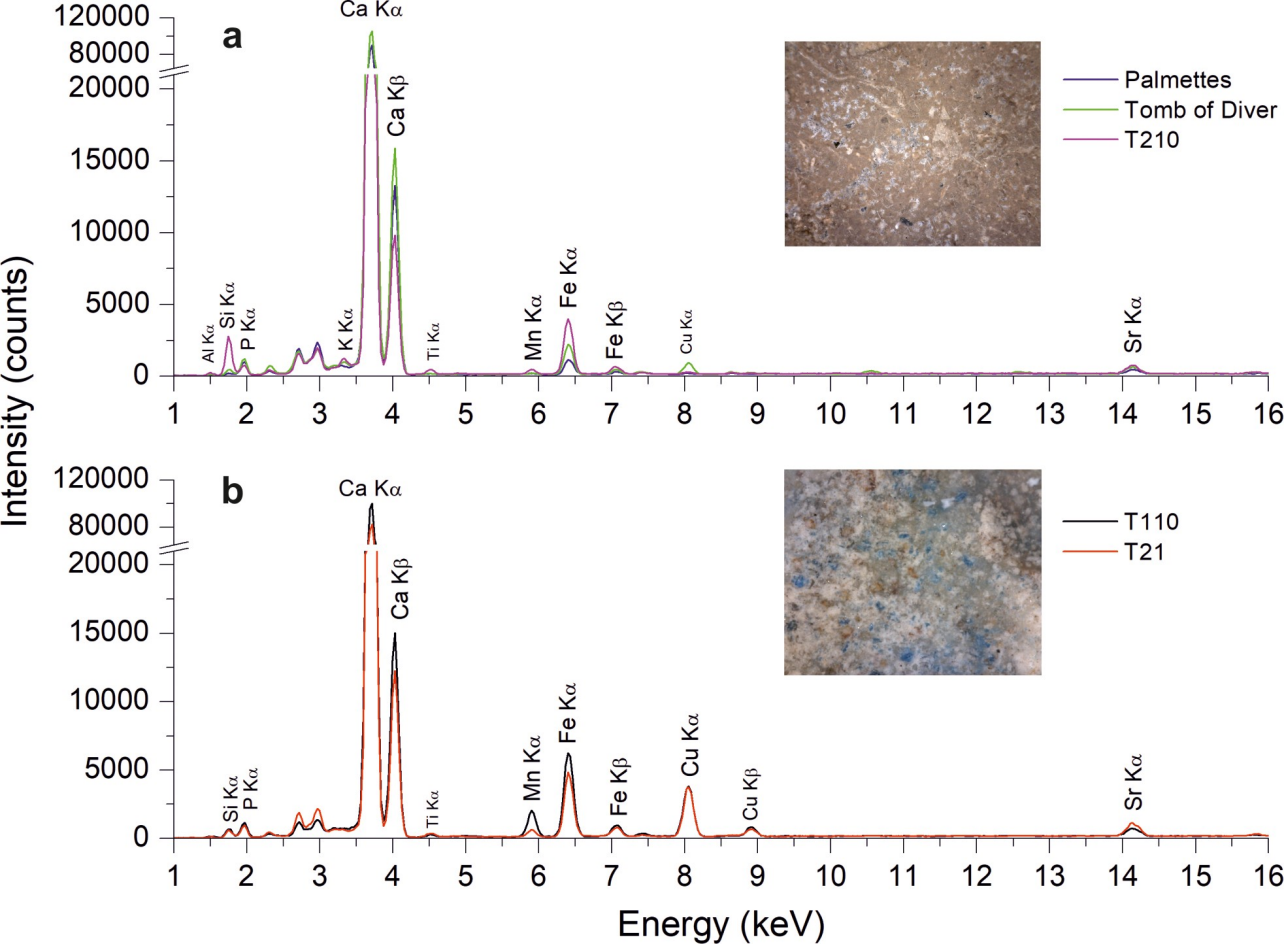
Chemical composition of green pigments. (a) XRF spectra of green pigments identified as green earths. (b) XRF spectra of green pigments identified as a mixture of Egyptian blue and iron-rich earths. Digital microscope images of both typologies of green pigments are also reported.

*Green pigments*. On green pictorial layers, two main typologies of pigments have been observed, mainly discriminated on the basis of their compositional features (Fig 7). The first type of green pigment was found in the Tomb of the Palmettes, in the tomb T210 and in the Tomb of Diver. Green paint used for decorating the former tombs is characterised by the presence of iron along with calcium in XRF spectra (Fig 7A). ER-FTIR also evidenced the presence of peaks in the spectral range between 3700 and 3500 cm^-1^ (OH stretching vibration) as well as the broad band between 1200 and 900 cm^-1^ (Si-O-Si stretching vibration) attributable to the presence of silicates. Such evidences suggest the use of green earths, natural pigments composed by hydrated Fe-rich silicates (e.g. glauconite, celadonite, chlorite) that give the green colour to the deposit [40–42], whose main supplying areas were located near Verona, Cyprus and Spain [32].

The second typology of green pigments (Fig 7B) was observed on the tombs T21 and T110, in correspondence of decorations depicting the leaves. On both tombs, these figurative details are characterized by a high absorption of visible light and VIL region (Fig 6C and 6D) attributable to cuprorivaite added in the pictorial mixtures. Differently, none of the green pictorial layers of the other investigated tombs appeared VIL active. Indeed, only the T21 and T110 green layers are characterised by the presence of higher copper content along with calcium and iron (Fig 7B) in the XRF spectra. Moreover, compositional features provided by FORS analyses confirm the use of Egyptian Blue pigment, likely mixed with another colour for obtaining the green shades. As a matter of fact, a mixture of Egyptian blue with iron-based earths can be invoked. In fact, the addition of the Egyptian blue to iron-rich earths to have brighter colour was a common practice, also in a later period among the Roman mural painters [32,33,41].

Moreover, a mixture of Egyptian blue with black pigments (carbon- and/or iron-based pigments) and iron-rich earths was already detected during previous analyses on painted slabs form Paestum [13–15], whereas the use of green earths was here observed for the first time.

**Fig 8:**
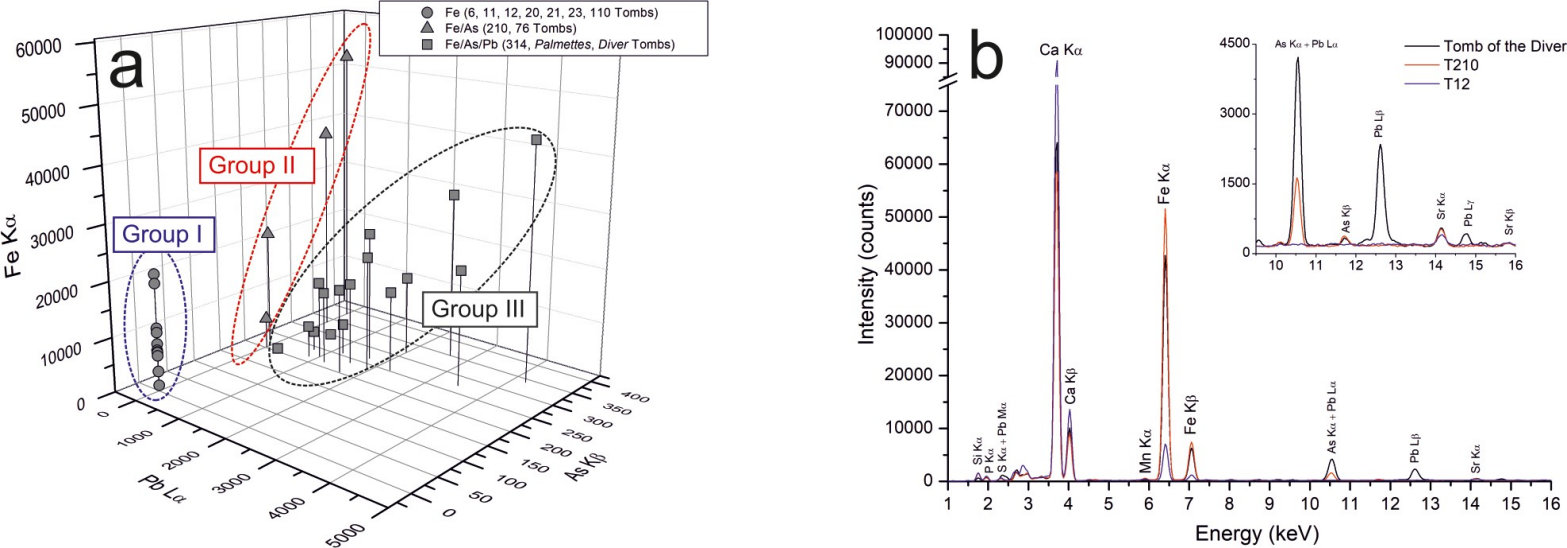
Chemical composition of red pigments. Fe-As-Pb XRF intensity plot for red pigments in which three compositional groups can be distinguished (Group I: red pigments composed of iron oxides -Tombs 6, 11, 12, 20, 21, 23 and 110-; Group II: iron-based pigments containing arsenic traces -Tombs 76 and 210-; Group III: iron-based pigments containing arsenic and lead traces -Tomb 314, Tomb of the Palmettes and Tomb of the Diver-). (b) Representative XRF spectra (Tomb 23 for Group I, Tomb 210 for Group II, Tomb of the Diver for Group III).

*Red pigments*. The red layers, both in darker and lighter shades (flesh tones), have different compositional characteristics that permitted individuating three different groups (Fig 8A). Red paints observed in T6, T11, T12, T20, T21, T76, T109 and T110 tombs are characterised by the presence of iron, and low counts of aluminium, silicon, potassium, manganese and sulphur, as highlighted by XRF (Fig 8B). FORS positively identified iron-based pigments in almost all the tombs whereas Raman spectra acquired on Palmettes, T12, T20, T21, T76, T110 red and pink layers showed the bands at ca. 410, 498 and 612 cm^-1^ imputable to hematite pigment (Fe_2_O_3_) according to literature [43–45].

Iron(III) oxide is the principal colouring matter in red ochre, the most widespread and used red pigment in the past [32]. Classical sources (Pliny, Vitruvius, Theophrastus) reported that the best red ochre was generally imported from Anatolia (i.e. *sinopia*) but such pigment was also obtained by grinding, sieving and burning a natural red earth containing iron oxides/hydroxides and other silicates for achieving the intense red colour [46]. In the past such red earths were generally imported [32] but it should be noted that possible exploitation sources also outcropped near Paestum in the Apennine sequences of the Salerno province [26]; thus, their exploitation in the past for the production of this red pigment cannot be excluded.

A second compositional group was constituted by red pigment of the tombs T76 and T210; here, red hues show, along with iron and calcium, the presence of arsenic traces that suggests the use of different red pigments (Fig 8B). Although FORS, ER-FTIR and Raman did not show significant spectral evidence, the compositional characteristics could be indicative of a mixture of pigments, likely constituted by a Fe-rich based pigment (e.g. red ochre) and low amount of arsenic-based pigment, used on red layers both in lighter and darker shades. Finally, in addition to iron and arsenic traces, the Tomb of the Diver, the Tomb of the Palmettes and the Tomb 314, the presence of lead-based pigment has been detected in the red hues (Fig 7B).

*Yellow pigments*. As observed for other pigment hues, also for yellow paints different compositions were found. In particular, yellow decorations in the Tomb of the Diver (south slab) and in Tomb T6 (north slab) contain arsenic along with calcium. Although the use of arsenic-based pigment could be invoked, but the univocal identification of this yellow pigment requires further analytical investigations to verify the possible use of an arsenic sulphide phases, widely used as pigments since ancient times [32].

**Fig 9:**
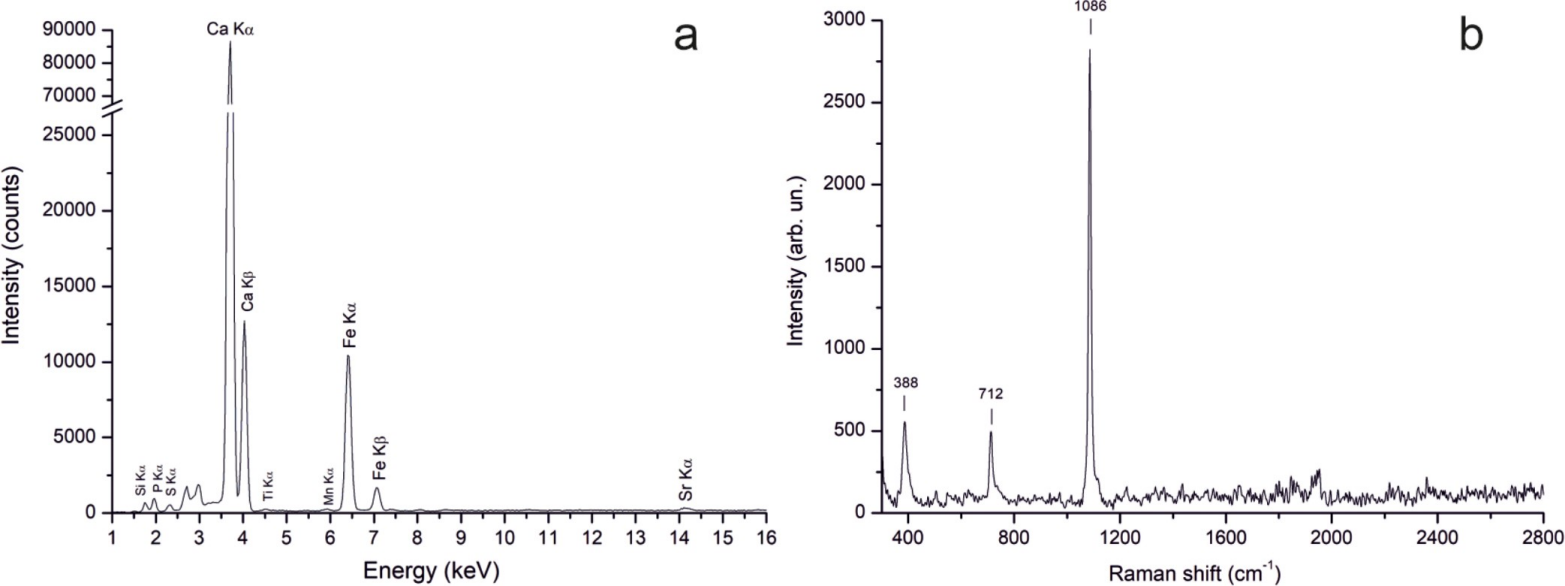
Yellow pigment identified as yellow ochre. (a) XRF spectrum. (b) Raman spectrum. Tomb T12, short slab.

By contrast, the yellow layers analysed in the other tombs (Andriuolo T12 and T20 Tombs, Phase III) are instead made of iron-based pigments. XRF analyses showed the presence of iron along with calcium (Fig 9A) and FORS analyses suggest the use of a pigment based on iron hydroxides (yellow ochre). Moreover, the Raman spectra, showed the presence of a peak at 388 cm^-1^, attributable to goethite [43] (Fig 9B). Goethite is the main constituent of yellow ochre, pigment based on iron hydroxides, clay minerals and other silicates as accessory phases, deriving from the direct alteration of iron-rich deposits, whose main supply areas of yellow ochre were located in Greece, Italy, Gaul and Turkey [32]. Its use for decorating other painted slabs in Paestum was also detected [13–15].

*Black pigments*. Finally, the black decorations have been analysed. XRF spectra did not provide energetic characteristic lines that allow identification of the chromophore (Fig 5A). However, the presence of weak Raman bands at 1596 and 1320 cm^-1^ permitted the identification of carbon black pigment [26,47]. The latter can be considered the most common type of black pigment described by historical literature, usually obtained from the combustion of coal, wood, oil or other fuel able to produce soot [36].

Interestingly, the same results have been obtained using a statistical approach that automatically compares the whole XRF spectrum of each measurement along with the age and the necropolis of provenance. The resulting dendrogram (S2 Fig) allowed distinguishing two main clusters with at least seven sub-groups coherent with chemical composition of the used pigments. In particular, pigments that mainly suffer of the carbonate-bearing substrate were clustered in Group 1 and 2, Mn-bearing black patinas strongly characterise the Gaudo, Andriuolo and Santa Venera tombstones (Group 3) and Cu-based pigments and the restoration products characterise Groups 5, 6 and 7 (S2 Fig). It is worth to note that the Group 4 clusters the Tombs of the Diver and the Tomb of the Palmettes, confirming the compositional similarities in the implemented palette.

### Mortar-based supports

#### Textural, mineralogical and compositional features of mortars

The investigation of technological changes in the considered time-span passed through the study of mortar-based supports, analysed by micro-destructive mineralogical and geochemical techniques. The sampling also involved coating mortars from the temples preserved in the archaeological site of Paestum, dated back to the Phase I, with the aim of evaluating eventual similarities with the decoration supports of painted tombs and that could suggest a standardization of production techniques.

From a macroscopic point of view, the analysed samples show high variability. With the exception of sample T21, all mortars show a multi-layered structure (at least two layers, namely *intonachino* and preparation layer); the *intonachino* has a variable colour (white, light grey or light brown), containing often rounded carbonate inclusions (high B/A ratio). The preparation layer, always white-beige colour, has a very high B/A ratio and is often covered by the pictorial layer. Most of the samples are tenacious, except for the samples T210 and T210L, which instead are very tenacious, and for the samples T21, T76 and T110C_1, almost crumbly. Rare lumps were observed in all samples.

**Fig 10:**
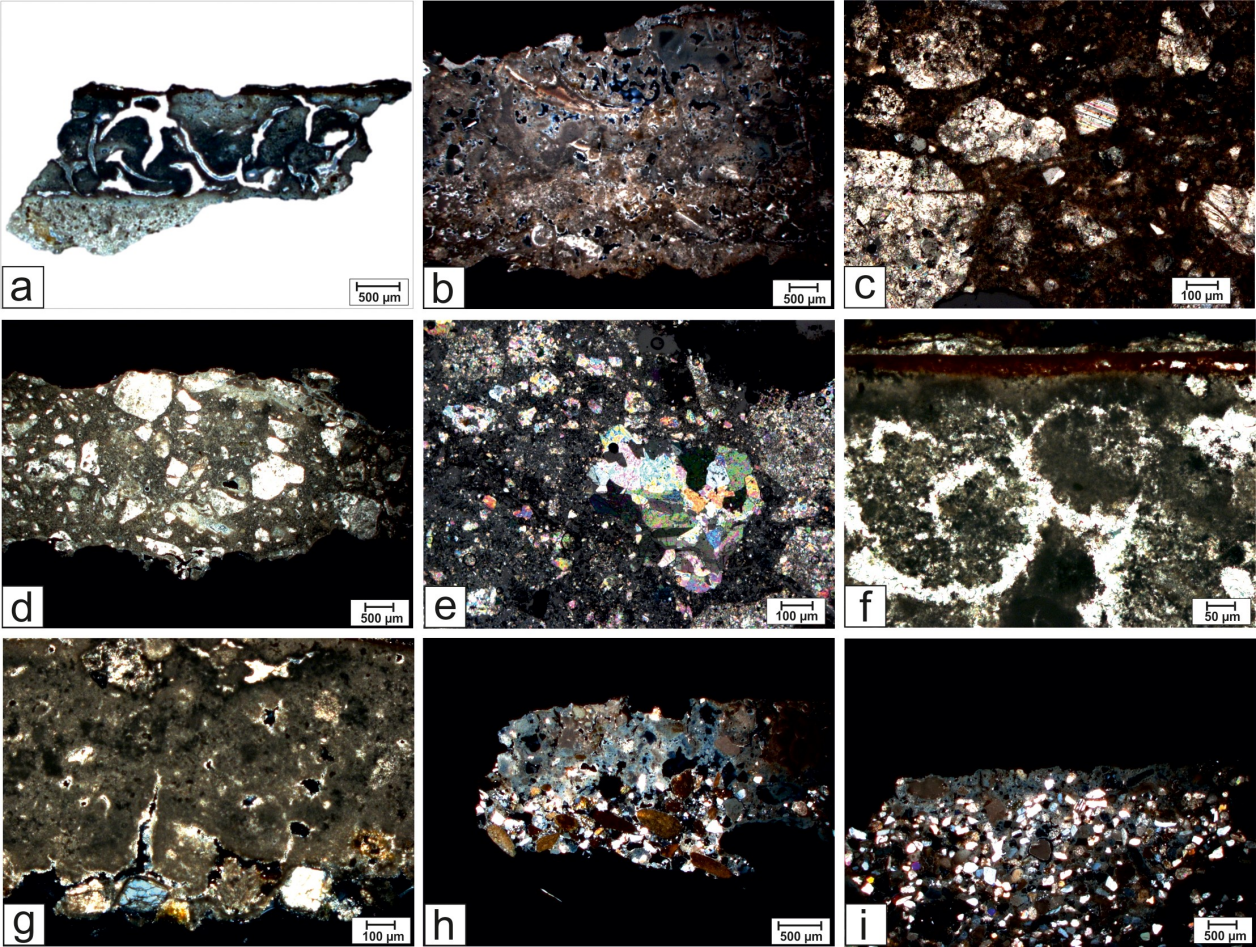
Representative microphotographs of mortar samples. (a) Sample PAL2R, microscopic features of mortar that allowing recognizing two different layers, plane polarized light, 1x. (b) Sample T12, microcrystalline binder containing fragments of fossils shells, crossed polars, 1x. (c) Sample TN4, calcite-bearing aggregate, crossed polars, 5x. (d) Sample BS2, aggregate-bearing mortar, crossed polars, 1x. (e) Sample TN3, aggregate composed by marble dust, crossed polars, 5x. (f) Sample T109C, pictorial layer, crossed polarized light, 10x. (g) Sample T21, aggregate in the inner layer of the mortar, crossed polars, 5x.(h) Sample T23, aggregate in the inner layer of the mortar, crossed polars, 1x. (i) Sample T76, aggregate in the inner layer of the mortar, crossed polars, 1x.

**Fig 11:**
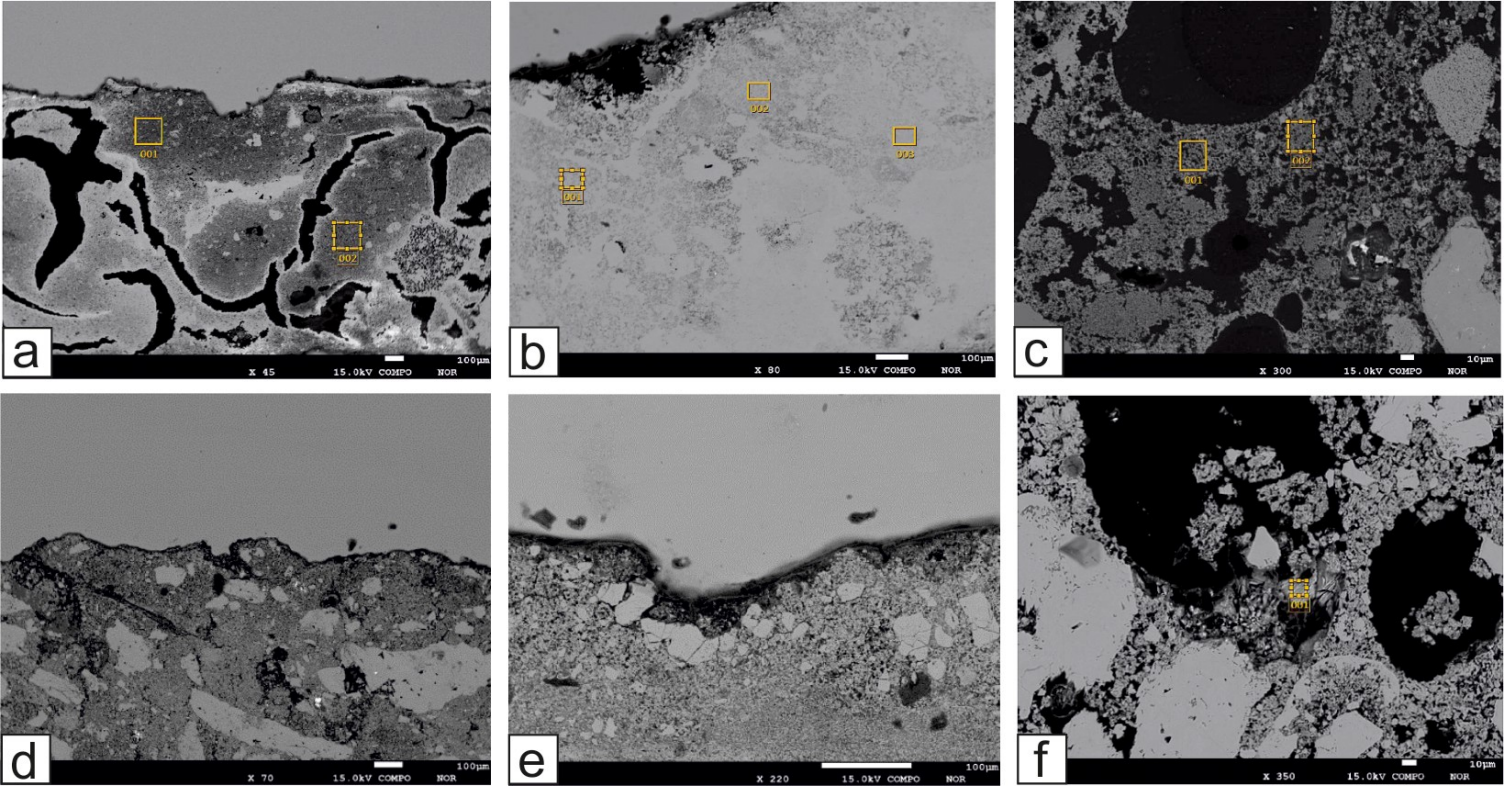
SEM images of representative samples. (a) Fractured binder of the outer layer of sample PAL2. (b) Microcrystalline binder of sample T109. (c) Binder of sample T76L. (d) Outer area of gypsum accumulation in the aggregate-bearing mortar TN1. (e) External surface of sample TN2. (f) Gypsum crystals in a pore of sample TN5. Yellow boxes indicate the areas of EDS analysis.

OM analyses showed that the samples of mortar-based supports of painted tombs are constituted of micritic and/or cryptocrystalline binders (Fig 10A and 10B; Fig 11A and 11B), constituted essentially of calcite. These are affected by advanced secondary recrystallization phenomena, effacing the original characteristics of the material. Moreover, a deep fracturing affects most of the analysed samples, likely as a consequence of the volumetric contractions (during the setting and hardening processes) due to the absence of the aggregate [48].

In detail, two distinguishable layers of plaster were identified in different samples (TUF4, BS1, PAL1, PAL2R, PAL2N) thanks to the appearance of the binder, its compactness or fracturing degree (Fig 10A). In TUF1, TUF2, TUF3, TUF4, PAL1, PAL2R, PAL2N, T11C, T11L, T21, T109, T210L, T314C, T314L, BS1, the presence of aggregate was not detected. On the contrary, fragments of fossils (Fig 10B) and calcite fragments with even coarse grain size were observed in T210, T12, T20, T110C, TN1, TN3, TN4, TN5, BS2 and BS3 (Fig 10C). In particular, the fragments reach ca. 1 mm in BS2 and BS3 (Fig 10D). TN2 is the only sample to be constituted exclusively by marble dust fragments (Fig 10E).

Moreover in many samples (TUF1, TUF2, TUF3, TUF4, PAL2R, PAL2N, T11C, T11L, T12, T20; T109, T110, T210) traces of the overlying pictorial layer are visible; its thickness is variable from 12 to 60 ***μ***m and pigments are absorbed in it (Fig 10F). These layers were analysed in more detail by the means of the dedicated techniques already discussed.

The lime-rich nature of the samples is also confirmed by spectroscopic and thermal analyses. ATR-FTIR spectra (S3 Fig; S1 Table), in fact, show its typical infrared bands at ca. 2515, 1795, 1420, 875 and 713 cm^*-1*^ [49]. In this samples the calcite content ranges from 70.0 to 94.0% (S2 Table), as quantified by thermal analyses by evaluating the weight loss between 600 and 850°C. In this thermal range, in fact, carbonate decomposes with an endothermic reaction, observed in all samples in-between 760 and 820°C, during which CO_*2*_ is released, as also detected by EGA (S2 Table).

The aggregate was observed only in the samples T21, T23 and T76L, in which it was added to the binder in the innermost layers of the mortar-based supports of the painting layer.

In T21 the aggregate is composed of a few crystals of quartz and plagioclase (Fig 10G). In T23 fragments of shales, crystalline rocks, minerals such as quartz, biotite, muscovite, feldspar, altered plagioclase and opaque minerals have been identified. The average size of the fragments is coarse (about 1 mm) (Fig 10H). On the other hand, the aggregate fraction observed in the inner layer of sample T76L consists of calcite, quartz, biotite, muscovite, pyroxenes, feldspars, fragments of both carbonate and crystalline rocks and fossil shells (Fig 10I). It underlies a layer of yellowish binder on which the pictorial layer was absent.

Thus, the presence of the aggregate fraction, as well as the typology of the fragments inside, permitted a distinction between the plasters that, however, lies outside from the chronological subdivision in the three phases but likely depends on the different functions that samples had. For samples T21, T23 (Phase III) and T76L (Phase II), in fact, a multi-layer technology was adopted, preparing at least two layers (an inner layer containing rounded aggregate and an outer layer only composed of binder); the addition of aggregate in the innermost layer, likely in contact with slab surfaces in travertine, was probably due the creation of a workable mixture that permitted a planar correction of the slab roughness whereas the outermost layer only composed of binder was likely needed for creating a white supporting medium for the painting [50].

The plasters of the slabs placed as decoration of the tombs and the coating mortars of the temples show similar characteristics with no clear changes relative at the three chronological phases. The only criterion that can be partially applied is the presence of the aggregate fraction in some samples and its absence in others, which could suggest both a different origin of the raw materials or another kind of executive technique.

EMPA-EDS microanalysis provided morphological and compositional information on the binder. The first suggests how the binder is strongly recrystallized and degraded in many areas; moreover, common phenomena of secondary calcite formation were observed in the pores and in the fractures as previously in OM (Fig 11A–11C).

The chemical data are reported in Tables 3 and 4 and supply the main composition in terms of major elements and the degree of hydraulicity of the binder [51]. These kind of information are generally crucial to identify the raw materials used in the production of the historic lime mortars [51–54]. In particular, the obtained data allowed calculating the Vicat's Index [55] also referred as the Hydraulicity Index (HI), expressed as the ratio Al_*2*_O_*3*_+SiO_*2*_+Fe_*2*_O_*3*_/CaO + MgO (Tables 3 and 4).

**Table 3:** Average values of major element concentrations (wt%) of mortars from tomb slabs, determined through EMPA-EDS analysis of binder in mortar samples. Hydraulicity Index HI = (Al_2_O_3_ + SiO_2_) / (CaO+MgO). Legend type: (A) aerial lime; (AH) average hydraulic lime.

Period	Tomb	ID	Na_2_O	MgO	Al_2_O_3_	SiO_2_	ClO	CaO	Total	Al_2_O_3_ + SiO_2_	CaO + MgO	HI	Type
**Phase I**	**Tomb of the Diver**	TUF1_01	-	0.07	0.25	1.02	0.78	97.89	100.00	1.27	97.96	0.01	A
		TUF1_02	-	0.28	0.37	0.78	0.75	97.82	100.00	1.15	98.10	0.01	A
		TUF2_01	-	0.58	0.63	1.14	-	97.65	100.00	1.77	98.23	0.02	A
		TUF2_02	-	0.62	-	1.22	-	98.17	100.00	1.22	98.79	0.01	A
		TUF3_01	-	0.25	0.31	0.51	0.77	98.15	100.00	0.82	98.40	0.01	A
		TUF3_02	-	0.23	0.35	0.57	1.04	97.81	100.00	0.92	98.04	0.01	A
		TUF4_01	-	0.52	0.33	1.15	1.63	96.37	100.00	1.48	96.89	0.02	A
		TUF4_02	-	0.78	0.19	1.43	1.36	96.25	100.00	1.62	97.03	0.02	A
	**Tomb of the Palmettes**	PAL1_1	-	0.26	0.36	0.41	0.50	98.47	100.00	0.77	98.73	0.01	A
		PAL1_2	-	0.33	0.22	0.36	0.51	98.58	100.00	0.58	98.91	0.01	A
		PAL2_1	-	0.91	-	1.00	0.88	97.21	100.00	1.00	98.12	0.01	A
		PAL2_2	0.09	0.97	-	1.73	0.88	96.33	100.00	1.73	97.30	0.02	A
		PAL2N_1	0.72	1.33	0.19	0.97	0.79	96.00	100.00	1.16	97.33	0.01	A
		PAL2N_2	0.53	1.03	0.30	0.96	1.64	95.54	100.00	1.26	96.57	0.01	A
		PAL2R_1	0.54	0.15	0.56	0.55	3.24	94.96	100.00	1.11	95.11	0.01	A
		PAL2R_2	-	0.36	0.69	1.28	2.25	95.42	100.00	1.97	95.78	0.02	A
	**Tomb 210**	T210_1	0.24	0.45	2.05	12.42	0.99	83.85	100.00	14.47	84.30	0.17	AH
		T210_2	0.12	0.57	2.84	12.16	0.67	83.64	100.00	15.00	84.21	0.18	AH
**Phase II**	**Tomb 76**	T76C_1	0.05	1.58	0.95	5.83	0.45	91.14	100.00	6.78	92.72	0.07	A
		T76C_2	0.05	1.65	0.64	4.39	0.47	92.81	100.00	5.03	94.46	0.05	A
		T76L_1	0.43	1.08	0.47	3.48	2.26	92.28	100.00	3.95	93.36	0.04	A
		T76L_2	0.49	1.31	0.81	4.81	2.21	90.38	100.00	5.62	91.69	0.06	A
	**Tomb 109**	T109_1	-	1.36	0.54	4.41	-	93.70	100.00	4.95	95.06	0.05	A
		T109_2	-	1.71	0.83	5.03	-	92.43	100.00	5.86	94.14	0.06	A
**Phase III**	**Tomb 12**	T12_1	-	0.76	1.27	18.34	1.45	78.18	100.00	19.61	78.94	0.25	AH
		T12_2	-	0.66	1.22	19.65	1.41	77.06	100.00	20.87	77.72	0.27	AH
	**Tomb 21**	T21_1	0.23	1.10	4.09	11.69	1.57	81.32	100.00	15.78	82.42	0.19	AH
		T21_2	0.25	1.27	4.07	11.70	1.83	80.88	100.00	15.77	82.15	0.19	AH
	**Tomb 23**	T23_1	0.22	0.40	0.21	16.16	0.50	82.51	100.00	16.37	82.91	0.20	AH
		T23_2	0.35	0.60	1.23	12.61	1.10	84.11	100.00	13.84	84.71	0.16	AH

**Table 4:** Average values of major element concentrations (wt%) of mortars from the Neptune temple, determined through EMPA-EDS analysis of binder in mortar samples. Hydraulicity Index HI = (Al_2_O_3_ + SiO_2_) / (CaO+MgO). Legend type: (A) aerial lime; (AH) average hydraulic lime.

Period	Tomb	ID	Na_2_O	MgO	Al_2_O_3_	SiO_2_	P_2_O_5_	SO_3_	ClO	K_2_O	CaO	Total	Al_2_O_3_ + SiO_2_	CaO + MgO	HI	Type
**Phase 1**	**Neptune Temple**	TN1_02	-	2.59	-	1.37	3.27	0.56	2.60	-	89.61	100.00	1.37	92.20	0.01	A
		TN1_03	0.90	1.92	-	0.85	1.77	0.64	3.49	-	90.43	100.00	0.85	92.35	0.01	A
		TN2_01	0.56	2.18	0.16	1.06	0.19	0.36	1.78	0.28	93.43	100.00	1.22	95.61	0.01	A
		TN2_02	1.11	1.55	0.34	1.24	0.37	0.79	1.93	0.58	92.09	100.00	1.58	93.64	0.02	A
		TN2_03	0.98	1.58	0.30	1.52	0.19	0.63	3.60	0.38	90.82	100.00	1.82	92.40	0.02	A
		TN2_04	0.66	1.65	0.21	1.67	0.25	0.61	4.37	0.31	90.27	100.00	1.88	91.92	0.02	A
		TN2_05	0.96	1.38	0.29	1.17	0.38	0.33	6.13	0.49	88.87	100.00	1.46	90.25	0.02	A
		TN5_01	-	1.14	-	0.61	-	0.34	3.91	-	94.00	100.00	0.61	95.14	0.01	A

Compositional data suggest how the binder consists almost exclusively of calcite (CaCO_*3*_) with higher percentage in wt% of CaO in Phase I and II than in the Phase III, where the amount of SiO_*2*_ increases (Table 3). In the Phase I, CaO exceeds 95% in the plasters relative to Tomb of the Diver and Tomb of the Palmettes, whereas it stands at around 83% with an increase in SiO_*2*_ and Al_*2*_O_*3*_ in the T210 tomb. Equally, CaO reaches and exceed 90% in Phase II (Table 3). Plasters of the Phase III show a different composition, as clearly suggested by CaO content varying between 77 and 84% and SiO_*2*_ increasing up to 19% (Table 3). The low content of MgO (0.1–1.3 wt%) suggested the only presence of calcite as carbonate.

The coating mortars from the Neptune Temple, also dated back to Phase I, show a certain compositional affinity with the binders of the tombs of the same phase with an average amount of CaO around 90%, and although higher contents of MgO occur (until 2.59 wt%; Table 4), likely due to a slight enrichment in magnesia of carbonate used to produce lime.

Moreover, it should be noted that mortars from temples are characterized by the presence of ClO, P_*2*_O_*5*_, SO_*3*_ (Table 4), suggesting alteration phenomena because of their exposition to the open-air weathering conditions for centuries. In this regard, EMPA observations highlighted the presence of gypsum crystals in samples TN1 and TN5 (Fig 11D–11F).

The Hydraulicity Index (HI) (Tables 3 and 4) is starkly under 0.10, the threshold of the field for aerial limes, in almost all the plasters: HI is generally around 0.01–0.02 in the samples from the Tomb of the Diver, the Palmettes and the Neptune Temple; HI reaches 0.07 in the tombs of the Phase II (Table 3). On the contrary, the samples of the Phase III and the tomb 210 of Phase I fall in the field of the average hydraulic limes (0.16 < HI < 0.31; Table 3).

The chemical analysis and the HI of the binders suggest the probable use of two type of limestone for the preparation of the samples, namely pure and marly limestone. Even though it is important to consider the advanced process of recrystallization of the binder in the samples and their scarce state of conservation that could influence the original composition.

Conversely, compositional data collected by the analysis on the binders and, especially the determination of HI, suggested a certain variability over the time: the plasters of Phases I (Tomb of the Diver, Tomb of the Palmettes, Neptune Temple) and Phase II (Tomb 76 and Tomb 109) were made with aerial lime, whereas Tomb 210 and the tombs of the Phase III (Tomb 12, Tomb 21, Tomb 23) with average hydraulic lime. This could probably suggest a difference in the raw materials used, respectively a purer and a marly limestone.

#### Alteration and degradation of mortar samples

As detected by EMPA-EDS analyses, coating mortars from temples are characterised by the presence of ClO P_*2*_O_*5*_ and SO_*3*_ that suggest alteration phenomena, expected in samples exposed to the weathering conditions for centuries. Other evidences of alteration phenomena were also provided by spectroscopic and thermal analyses. Thermal analyses, in fact, showed an exothermic peak at ca. 330°C (S2 Table) corrsponding to an emission of CO_*2*_ in EGA, due to the decomposition of organic matter, likely depositated on the mortars surfaces; moreover, an intense endothermic reaction at ca. 130°C was observed in the samples TN2 and TN5 (S2 Table). ATR-FTIR spectroscopy confirmed that this latter phenomenon can be attributed to the dehydration of gypsum, as indicated by the infrared bands at ca. 3540, 3517, 3400, 1681, 1619, 1115, 670, 600 cm^*-1*^ [27] (S1 Table; S3 Fig), occurring in the sample as harmful product formed as a consequence of sulphation phenomena [31]. SEM observations (Fig 11D–11F) highlighted the presence of gypsum crystals also in the sample TN1.

## Conclusions

The multi-analytical techniques used for the characterisation of the painted tomb slabs and temples from the archaeological site of Paestum revealed significant information on raw materials and technological features. Red and green identified pictorial mixtures have proven to be important markers for a systematic comparative analysis used pigments in the three different chronological phases. Moreover, the typologies of mortars, allowed differentiating the executive techniques, highlighting the change in choices and technological skills of artisans since the first period of the frescoed tombs tradition at Greek colony.

The results shed light on the decorative techniques and pigments adopted from the Hellenistic to Lucan period. In particular, interesting similarities in red and grey-green layers chemical composition and mortars technical features have been highlighted between the Tomb of the Diver and the Tomb of the Palmettes, which represented one of the main archaeological issues asked by the archaeologists supporting the belonging of these two coeval tombs to a still unexplored local artisanal tradition at the Greek city in 500–475 BC.

Valuable information has been obtained by the compositional data of pigments that permitted the identification of the palette used for decorating the slabs. The use of calcite, red and yellow ochres, green earths, Egyptian blue and black carbon was revealed by *in situ* measurements. Moreover, the possible adoption of As-rich yellow and red pigments should be verified, representing, along with the presence of lead in the red hues, one of the discriminative features for the Tomb of the Diver and Tomb of the Palmettes.

The use of the Cu-based pigment in the green pictorial layers of the third period (post 300 BC) suggests a greater availability of this precious synthetic pigment starting from this time. The main evidence is related to the high similarity, in terms of pigment palette, between the Tomb of Diver and Tomb of the Palmettes, which shown similar chemical composition for each analysed colour. However, archaeometric evidences highlighted that they are quite different from the painted tombs of the Lucan period, supporting the archaeological hypothesis based on a stylistic point of view.

In order to better understand if such similarities can be also observed in the manufacturing of underlying plasters, analyses on micro-samplings (e.g. OM, EMPA-EDS, TG/DSC-FTIR(EGA), ATR-FTIR) have been carried out. Their features, as well as those of other public buildings (e.g. *Basilica*, temples), allowed deepening the knowledge on the production technology adopted form the end of the 6th and the first half of the 4th century BC in Paestum and also evaluating possible changings occurred during a time span of about two centuries.

Results of analyses on micro-sampling of the tombs shed light on the stratigraphic structure (from pictorial to preparation layers and supports) and mineralogical composition of samples, suggesting different technological choices used for the execution of the painted slabs, in particular during the Phase III.

The plasters of the tombs and of the temples show similar characteristics, highlighting no clear changes relative during the three chronological phases. The only criterion that can be partially applied is the presence of the aggregate fraction in some samples and its absence in others, which could suggest both a different origin of the raw materials or another kind a different of executive technique, depending on the different functions. Finally, a changing in the production technology of mortar-based supports has been verified thank to the hydraulicity index evaluation. The plasters of Phases I and Phase II were made with aerial lime, whereas the tombs of the Phase III (and the mortars of the tomb in addition to the T210 mortars, Phase II) with average hydraulic lime. This could probably suggest a difference in the raw materials used, respectively a purer and a marly limestone.

The archaeometric data suggest that the Tomb of the Diver could belong to a local artisanal tradition and therefore is neither Etruscan nor Greek, but first and foremost an expression of the local elite culture of Paestum. Furthermore, the data suggests that the expertise and technology used for the frescoed tombs originated in the field of temple architecture. Moreover, the analysis of plaster samples from the temples and the tombs suggests that the workshops were closely connected, or even the same.

## Supporting information

S1 Fig Analysed frescoed tombs. Images of the tombs from Andriuolo, Gaudo and Santa Venera necropolis analysed within the research project.
(PDF)

S2 Fig Statistical treatment of XRF data. Dendrogram obtained by statistical treatment of intensity XRF signal values, showing at least seven groups. Groups 1 and 2 mainly cluster white pigments (likely composed by predominant Ca and lower Al, Si, Fe and Sr) and black hues (likely here clustered since the impossibility of detecting carbon). The other colours here observed (red, yellow and green) thus contain the same elements (i.e. iron-rich earth pigments). The Group 3 is constituted of pigments of different tombs from Gaudo, Andriuolo and Santa Venera necropolis, characterised by the presence of external alteration black patinas containing manganese, which likely allowed isolating such a group. The Group 4 is composed of the Tomb of the Diver and the Tomb of the Palmette, confirming the similarities in the compositional features of pigments used for decorating them. Groups 5a and 6a are likely formed by pigments containing copper whereas the for the remaining groups (5b, 6b and 7a) a unique relation with the compositional Groups cannot be identified since the ubiquitous presence of chemical elements contained in the restoration products that likely influenced more than the constitutive elements of matrices and/or pigments.
(PDF)

S3 Fig Infrared spectra and thermal analysis curves. Representative ATR-FTIR spectra and TG-DSC-DTG curves of samples PAL2 (a,b), T76L (c,d) and TN2 (e,f).
(PDF)

S1 Table Infrared data. Infrared peaks obtained by ATR-FTIR analyses and vibrational assignments.
(PDF)

S2 Table Thermal analysis data. Weight-losses and enthalpy changes of analysed samples by STA analyses. Negative peaks observed on derivative thermogravimetric curves (DTG) are also reported.
(PDF)
