# Supplementary material for: The Tomb of the Diver and the frescoed tombs in Paestum (southern Italy): New insights from a comparative archaeometric study
Source: PLoS One. 2020 Apr 24;15(4):e0232375. doi: 10.1371/journal.pone.0232375 (PMC7182217; doi:10.1371/journal.pone.0232375)
Supplement: S1 Fig — Images of the tombs from Andriuolo, Gaudo and Santa Venera necropolis analysed within the research project. (PDF) [file pone.0232375.s001.pdf]

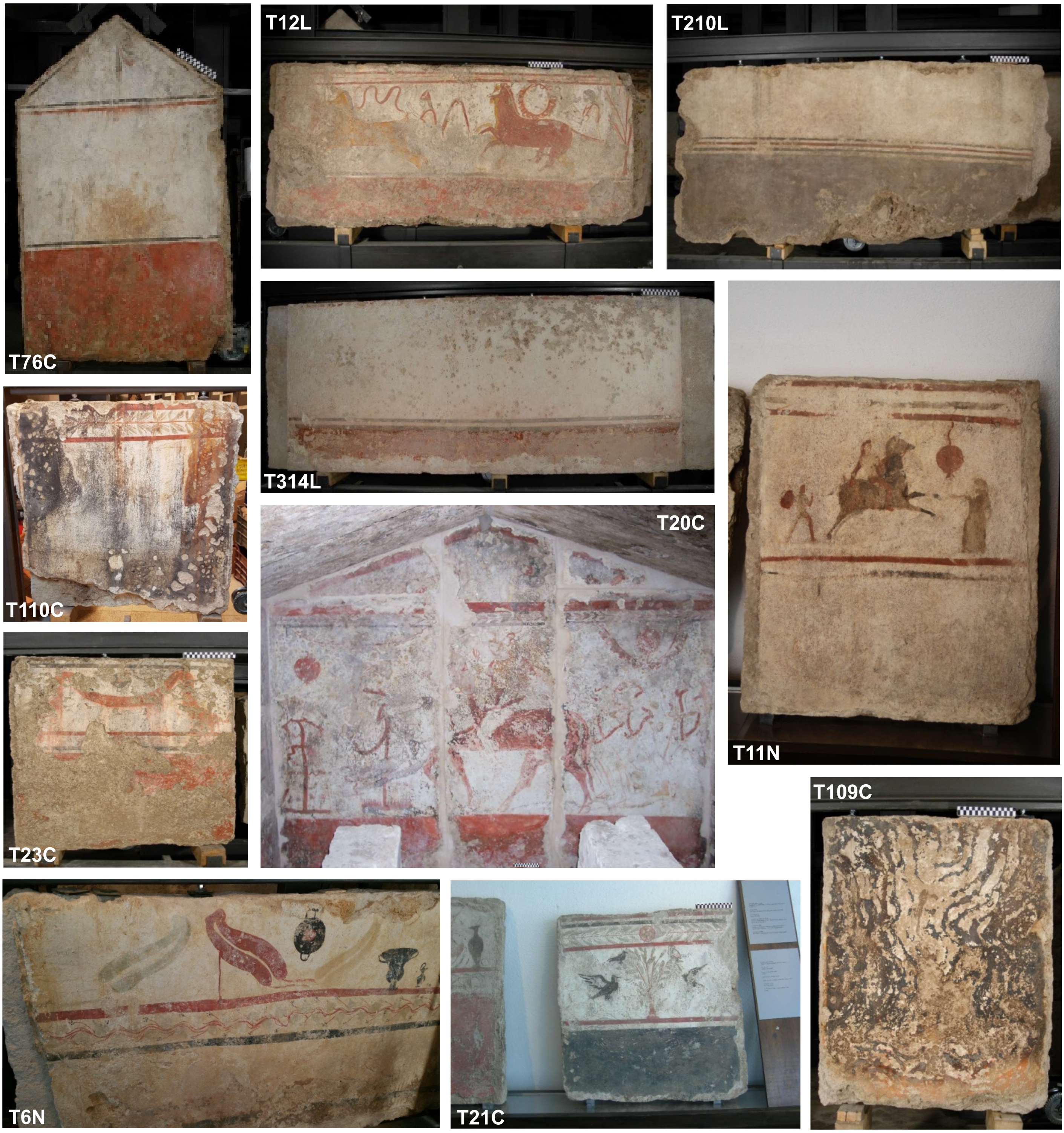

S1 Fig. Analysed frescoed tombs. Images of the tombs from Andriuolo, Gaudo and Santa Venera necropolis analysed within the research project.
