# Supplementary material for: The Tomb of the Diver and the frescoed tombs in Paestum (southern Italy): New insights from a comparative archaeometric study
Source: PLoS One. 2020 Apr 24;15(4):e0232375. doi: 10.1371/journal.pone.0232375 (PMC7182217; doi:10.1371/journal.pone.0232375)
Supplement: S2 Fig — Dendrogram obtained by statistical treatment of intensity XRF signal values, showing at least seven groups. Groups 1 and 2 mainly cluster white pigments (likely composed by predominant Ca and lower Al, Si, Fe and Sr) and black hues (likely here clustered since the impossibility of detecting carbon). The other colours here observed (red, yellow and green) thus contain the same elements (i.e. iron-rich earth pigments). The Group 3 is constituted of pigments of different tombs from Gaudo, Andriuolo and Santa Venera necropolis, characterised by the presence of external alteration black patinas containing manganese, which likely allowed isolating such a group. The Group 4 is composed of the Tomb of the Diver and the Tomb of the Palmette, confirming the similarities in the compositional features of pigments used for decorating them. Groups 5a and 6a are likely formed by pigments containing copper whereas the for the remaining groups (5b, 6b and 7a) a unique relation with the compositional Groups cannot be identified since the ubiquitous presence of chemical elements contained in the restoration products that likely influenced more than the constitutive elements of matrices and/or pigments. (PDF) [file pone.0232375.s002.pdf]

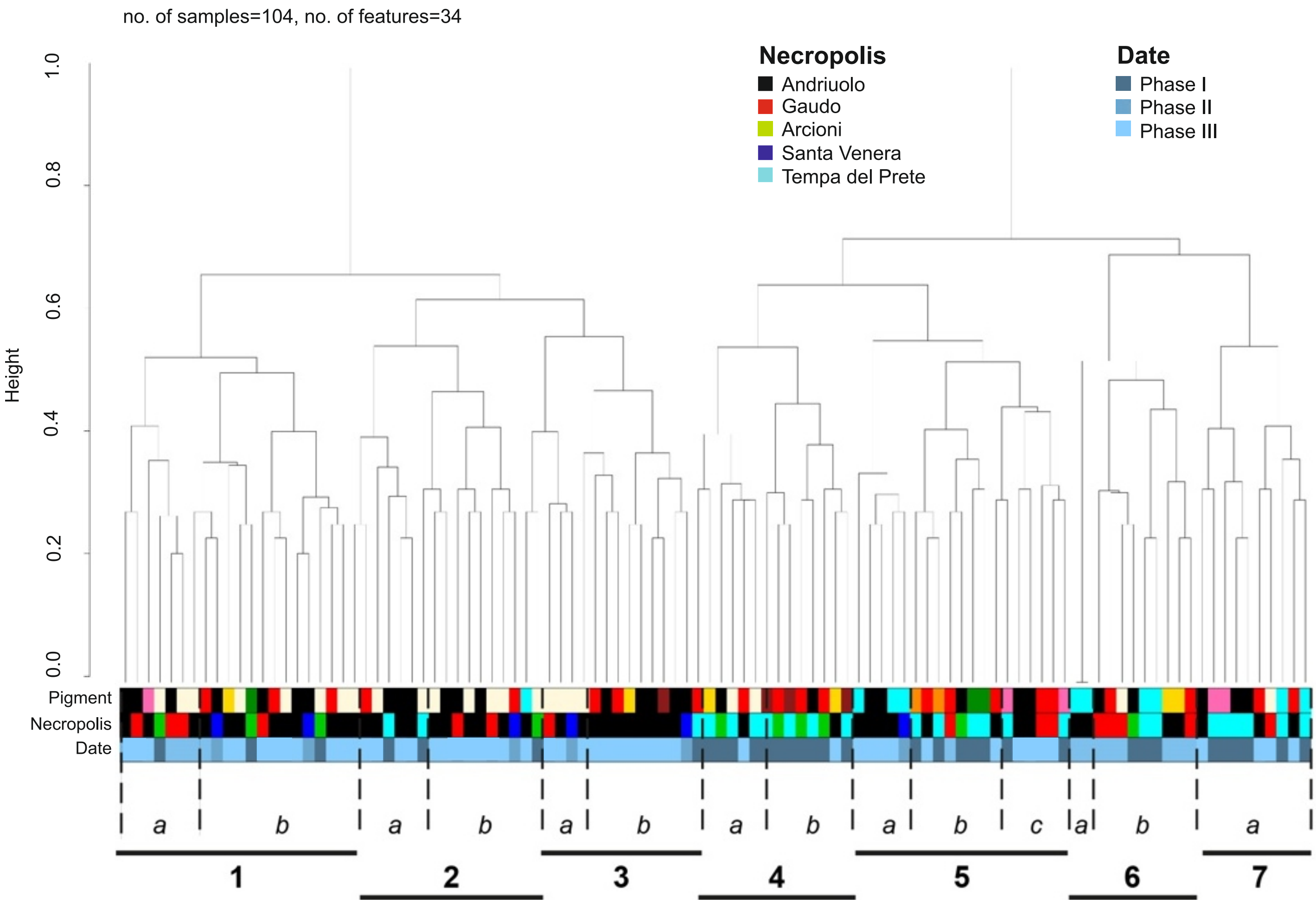

S2 Fig. Statistical treatment of XRF data. Dendrogram obtained by statistical treatment of intensity XRF signal values, showing at least seven Groups. Groups 1 and 2 mainly cluster white pigments (likely composed by predominant Ca and lower Al, Si, Fe and Sr) and black hues (likely here clustered since the impossibility of detecting carbon). The other colours here observed (red, yellow and green) thus contain the same elements (i.e. iron-rich earth pigments).
The Group 3 is constituted of pigments of different tombs from Gaudo, Andriuolo and Santa Venera necropolis, characterised by the presence of the external alteration black patinas containing manganese, which likely allowed isolating such a Group.
The Group 4 is composed of the Tomb of the Diver and the Tomb of the Palmette, confirming the similarities in the compositional features of pigments used for decorating them.
Groups 5a and 6a are likely formed by pigments containing copper whereas the for the remaining Groups (5b, 6b and 7a) a unique relation with the compositional Groups cannot be identified since the ubiquitous presence of chemical elements contained in the restoration products that likely influenced more than the constitutive elements of matrices and/or pigments.
