# Supplementary material for: The Tomb of the Diver and the frescoed tombs in Paestum (southern Italy): New insights from a comparative archaeometric study
Source: PLoS One. 2020 Apr 24;15(4):e0232375. doi: 10.1371/journal.pone.0232375 (PMC7182217; doi:10.1371/journal.pone.0232375)
Supplement: S3 Fig — Representative ATR-FTIR spectra and TG-DSC-DTG curves of samples PAL2 (a,b), T76L (c,d) and TN2 (e,f). (PDF) [file pone.0232375.s003.pdf]

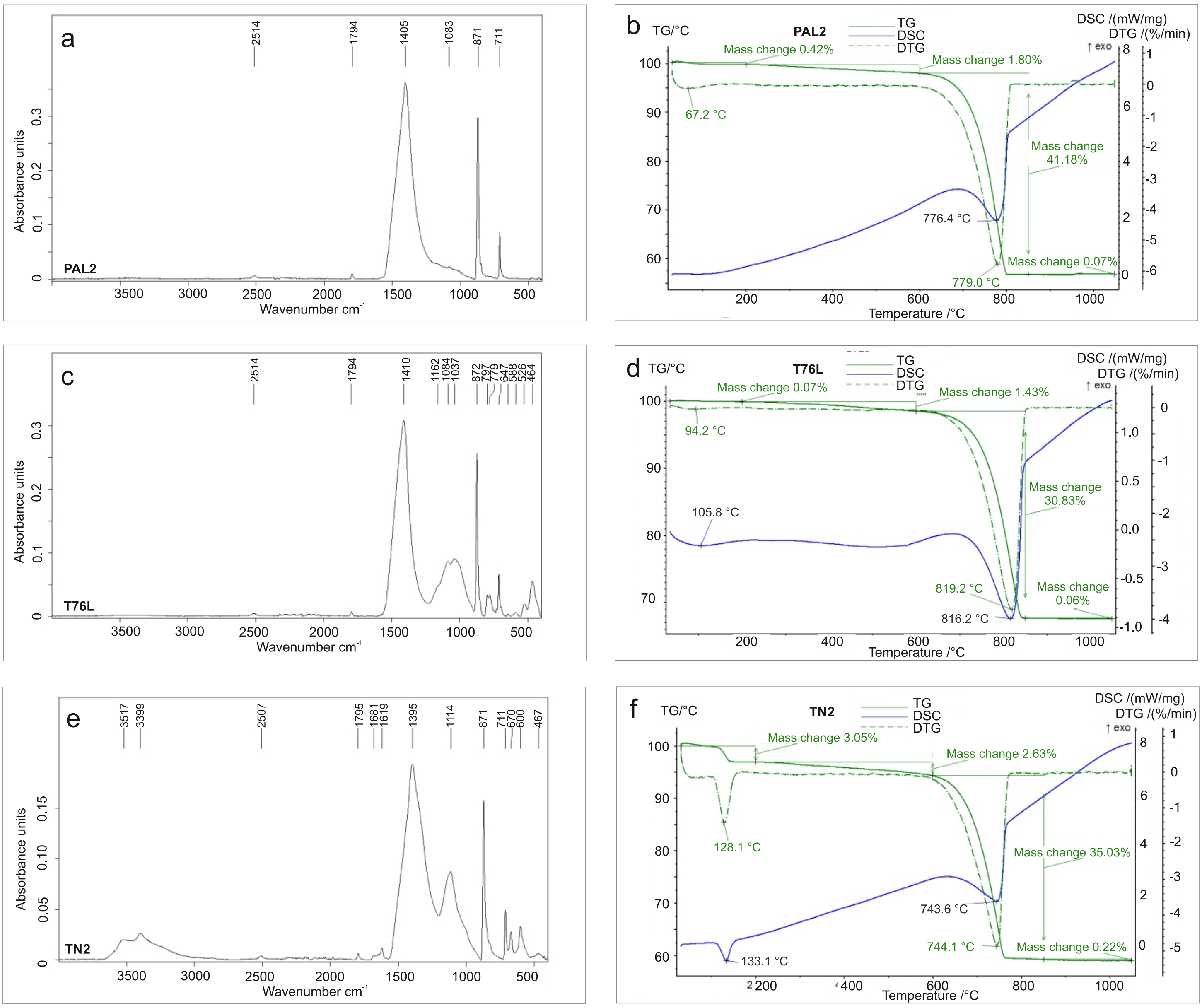

S3 Fig. Infrared spectra and thermal analysis curves. Representative ATR-FTIR spectra and TG-DSC-DTG curves of samples PAL2 (a,b), T76L (c,d) and TN2 (e,f).
