# Supplementary material for: The Tomb of the Diver and the frescoed tombs in Paestum (southern Italy): New insights from a comparative archaeometric study
Source: PLoS One. 2020 Apr 24;15(4):e0232375. doi: 10.1371/journal.pone.0232375 (PMC7182217; doi:10.1371/journal.pone.0232375)
Supplement: S1 Table — Infrared peaks obtained by ATR-FTIR analyses and vibrational assignments. (PDF) [file pone.0232375.s004.pdf]

| | BS1 | BS2 | PAL 1 | PAL 2 | T11 C | T11L | T12 | T20 | T20_ 1 | T21 | T76 C | T76L | T109 C | T110 C | T110 C_1 | T210 | T210 L | T314 C | T314 L | TN2 | TN3 | TN4 | TN5 | Vibrational assignments | Mineralogical phase |
|---|---|---|---|---|---|---|---|---|---|---|---|---|---|---|---|---|---|---|---|---|---|---|---|---|---|
| Infrared peaks ($cm^{-1}$) | | | | | | | | | | | | | | | | | | | | 3517 | | | 3541 | O-H stretching | Gypsum |
| | | | | | | | 3360 | 3408 | | 3349 | | | | | | | | | | 3399 | | 3360 | 3401 | O-H stretching | Water/Gypsum |
| | 2516 | 2514 | 2513 | 2512 | 2516 | 2515 | 2508 | 2515 | 2516 | 2512 | 2518 | 2514 | 2512 | 2517 | 2513 | 2516 | 2516 | 2516 | 2512 | 2507 | 2515 | 2509 | 2513 | Combination mode in $CO_3^{2-}$ | Carbonates |
| | 1794 | 1794 | 1795 | 1794 | 1794 | 1795 | 1795 | 1794 | 1795 | 1795 | 1794 | 1794 | 1794 | 1795 | 1795 | 1794 | 1794 | 1794 | 1795 | 1795 | 1795 | 1795 | 1795 | Combination mode in $CO_3^{2-}$ | Carbonates |
| | | | | | | | | | | | | | | | | | | | | 1681 | | | | O-H bending | Gypsum |
| | 1647 | 1658 | | | | | 1637 | 1650 | | 1647 | | | | | | | | | | | | 1641 | | O-H bending | Water |
| | | | | | | | | | | | | | | | | | | | | 1619 | | | 1620 | O-H bending | Gypsum |
| | 1406 | 1405 | 1406 | 1405 | 1406 | 1407 | 1412 | 1406 | 1406 | 1409 | 1408 | 1410 | 1407 | 1405 | 1406 | 1409 | 1410 | 1408 | 1407 | 1395 | 1404 | 1403 | 1407 | C-O asymmetric stretching | Carbonates |
| | 1160 | 1164 | | | 1160 | 1160 | | 1145 | 1161 | 1163 | 1162 | 1162 | | | | | | | | | 1163 | 1163 | | Si–O–Si stretching | Silicates |
| | | | | | | | | | | | | | | | | | | | | 1114 | | | 1123 | S-O asymmetric stretching | Gypsum |
| | | | 1083 | 1083 | | | 1082 | 1086 | | | 1080 | 1084 | 1083 | 1085 | 1084 | 1086 | 1084 | 1082 | 1082 | | | | | Si–O–Si stretching | Quartz/Silicates |
| | 1035 | 1022 | 1039 | | | 1037 | | | | 1023 | 1035 | 1037 | | | | | | | | | 1024 | 1026 | | Si–O–Si stretching | Silicates |
| | 872 | 871 | 872 | 871 | 872 | 872 | 872 | 872 | 872 | 872 | 872 | 872 | 872 | 872 | 872 | 872 | 872 | 872 | 872 | 871 | 872 | 871 | 872 | Out-of-plane bending in $CO_3^{2-}$ | Carbonates |
| | | | | | | | | | | 797 | 797 | 797 | | | | | | | 801 | | | | | Si–O–Si stretching | Quartz |
| | | | | | | | | | | 779 | 779 | 779 | | | | | | | | | | | | Si–O bending | Quartz |
| | 712 | 712 | 712 | 711 | 712 | 712 | 712 | 712 | 712 | 711 | 712 | 712 | 712 | 712 | 712 | 712 | 711 | 712 | 712 | 711 | 712 | 712 | 712 | In-plane bending in $CO_3^{2-}$ | Carbonates |
| | | | | | | | | | | | | | | | | | | | | 670 | | | 671 | S-O bending | Gypsum |
| | | | | | | | | | | | | 647 | | | | | | | | 600 | | | 602 | S-O bending | Gypsum |
| | | | | | | | | 604 | | | | 588 | | | | | | | | | | | | Al–O–Si bending | Silicates |
| | | 532 | | | | | 540 | | | 533 | | 526 | | | | | | | | | | | | Al–O–Si bending | Silicates |
| | 457 | 469 | 473 | | | 473 | 467 | 472 | | 464 | 468 | 464 | 468 | 469 | 461 | 466 | 465 | 459 | 462 | 467 | 447 | 459 | 472 | O-Si-O bending | Silicates |

**S1 Table. Infrared data.** Infrared peaks obtained by ATR-FTIR analyses and vibrational assignments.
