# Supplementary material for: The Tomb of the Diver and the frescoed tombs in Paestum (southern Italy): New insights from a comparative archaeometric study
Source: PLoS One. 2020 Apr 24;15(4):e0232375. doi: 10.1371/journal.pone.0232375 (PMC7182217; doi:10.1371/journal.pone.0232375)
Supplement: S2 Table — Weight-losses and enthalpy changes of analysed samples by STA analyses. Negative peaks observed on derivative thermogravimetric curves (DTG) are also reported. (PDF) [file pone.0232375.s005.pdf]

| | Dehydration | | | | Dehydroxylation and/or decomposition OC | | | | Decomposition of carbonates | | | | Polymorphic transformation, sintering behaviour and/or decomposition of sulphates | | | | L.O.I. (%) | R.M. (%) | $CaCO_3$% (600-850°C) |
|---|---|---|---|---|---|---|---|---|---|---|---|---|---|---|---|---|---|---|---|
| | <200°C | | | | 200-600°C | | | | 600-850°C | | | | >850°C | | | | | | |
| ID sample | ΔW (%) | DTG (°C) | DSC(a) (°C) | EGA | ΔW (%) | DTG (°C) | DSC(a,b) (°C) | EGA | ΔW (%) | DTG (°C) | DSC(a) (°C) | EGA | ΔW (%) | DTG (°C) | DSC (°C) | EGA | | | |
| **BS1** | 0.85 | 96.9 | - | - | 4.41 | 337.0 | 341 .2 (b) | $CO_2$ | 38.54 | 759.4 | 759 .6 | $CO_2$ | 0.18 | - | - | - | 43.98 | 56.02 | 87.5 |
| **BS2** | 0.73 | 86.8 | - | - | 3.28 | 341.0 | 341 .2 (b) | $CO_2$ | 39.42 | 796.9 | 797 .8 | $CO_2$ | 0.13 | - | - | - | 43.56 | 56.44 | 89.5 |
| **PAL1** | 0.03 | 85.1 | - | - | 2.63 | 385.6 | 385 .5 (b) | $CO_2$ | 40.42 | 783.0 | 784 .0 | $CO_2$ | 0.11 | - | - | - | 43.19 | 56.81 | 91.8 |
| **PAL2** | 0.42 | 67.2 | - | - | 1.80 | - | - | - | 41.18 | 779.0 | 776 .4 | $CO_2$ | 0.07 | - | - | - | 43.47 | 56.53 | 93.5 |
| **T11C** | 0.48 | 105.0 | - | - | 2.19 | - | 388 .0 (b) | $CO_2$ | 41.30 | 760.1 | 755 .5 | $CO_2$ | 0.02 | - | - | - | 43.99 | 56.01 | 93.8 |
| **T11L** | 0.00 | 111.1 | 112 .9 | - | 1.61 | - | - | - | 40.18 | 758.6 | 755 .6 | $CO_2$ | 0.11 | - | - | - | 41.90 | 58.10 | 91.2 |
| **T12** | 1.05 | 69.5 | 94 .7 | - | 4.52 | - | 352 .7 (b) | - | 33.46 | 786.5 | 785 .1 | $CO_2$ | 0.35 | - | - | - | 39.38 | 60.62 | 76.0 |
| **T20** | 0.44 | 118.8 | 119 .6 | - | 2.54 | - | - | - | 39.62 | 793.2 | 791 .2 | $CO_2$ | 0.17 | - | - | - | 42.77 | 57.23 | 89.9 |
| **T20_1** | 0.06 | 108.3 | - | - | 1.07 | - | - | - | 41.45 | 790.8 | 789 .1 | $CO_2$ | 0.02 | - | - | - | 42.60 | 57.40 | 94.1 |
| **T21** | 0.28 | 91.9 | 97 .3 | - | 1.96 | - | - | - | 30.85 | 792.9 | 790 .8 | $CO_2$ | 0.24 | - | - | - | 33.33 | 66.67 | 70.0 |
| **T76C** | 0.31 | 91.1 | 102 .5 | - | 2.39 | - | 378 .9 (b) | - | 34.64 | 807.9 | 804 .0 | $CO_2$ | 0.09 | - | - | - | 37.43 | 62.57 | 78.6 |
| **T76L** | 0.07 | 94.2 | 105 .8 | - | 1.43 | - | - | - | 30.83 | 819.2 | 816 .2 | $CO_2$ | 0.06 | - | - | - | 32.39 | 67.61 | 70.0 |
| **T109C** | 0.16 | 84.7 | 99 .7 | - | 2.49 | - | - | - | 39.29 | 800.7 | 797 .0 | $CO_2$ | 0.01 | - | - | - | 41.95 | 58.05 | 89.2 |
| **T110C** | 0.30 | 82.2 | - | - | 1.96 | - | - | - | 39.63 | 773.4 | 771 .2 | $CO_2$ | 0.16 | - | - | - | 42.05 | 57.95 | 90.0 |
| **T110C_1** | 0.75 | 90.0 | 93 .5 | - | 4.55 | 329.0 | 342 .9 (b) | - | 32.96 | 612 .0÷801 .7 | 798 .7 | $CO_2$ | 0.22 | - | - | - | 38.48 | 61.52 | 74.8 |
| **T210** | 0.50 | 72.0 | - | - | 4.89 | 382.9 | 389 .7 (b) | $CO_2$ | 34.97 | 772.7 | 769 .3 | $CO_2$ | 0.01 | - | - | - | 40.37 | 59.63 | 79.4 |
| **T210L** | 0.28 | 103.5 | 99 .1 | - | 3.00 | | - | - | 38.02 | 813.1 | 812 .4 | $CO_2$ | 0.08 | - | - | - | 41.38 | 58.62 | 86.3 |
| **T314C** | 0.24 | 89.0 | - | - | 8.24 | 348 .6÷590 .1 | 363 .6 (b) | - | 32.67 | 766.8 | 769 .1 | $CO_2$ | 0.30 | - | - | - | 41.45 | 58.55 | 74.2 |
| **T314L** | 0.04 | 77.3 | - | - | 3.40 | - | - | - | 36.78 | 636 .1÷786 .2 | 783 .2 | $CO_2$ | 0.03 | - | - | - | 40.25 | 59.75 | 83.5 |
| **TN2** | 3.05 | 128.1 | 133 .4 | - | 2.63 | - | - | - | 35.03 | 744.1 | 743 .6 | $CO_2$ | 0.22 | - | - | - | 40.93 | 59.07 | 79.5 |
| **TN3** | 0.25 | 94.2 | - | - | 2.77 | 350.6 | 352 .8 (b) | - | 39.64 | 784.4 | 784 .1 | $CO_2$ | 0.11 | - | - | - | 42.77 | 57.23 | 90.0 |
| **TN4** | 0.40 | 100.5 | 109 .4 | - | 2.64 | 333.0 | 339 .2 (b) | $CO_2$ | 40.25 | 808.3 | 804 .6 | $CO_2$ | 0.14 | - | - | - | 43.43 | 56.57 | 91.4 |
| **TN5** | 1.57 | 126.6 | 131 .8 | - | 2.06 | - | - | - | 37.68 | 777.3 | 775 .3 | $CO_2$ | 0.10 | - | - | - | 41.41 | 58.59 | 85.5 |

Legend: ΔW: weight loss (by TG); L.O.I.: Loss On Ignition; R.M: Residual Mass; OC: Organic Compounds; a: endothermic peak; b: exothermic peak.

**S2 Table**. **Thermal analysis data.** Weight-losses and enthalpy changes of analysed samples by STA analyses. Negative peaks observed on derivative thermogravimetric curves (DTG) are also reported.
